# Application of Economic Evaluation to Assess Feasibility for Reimbursement of Genomic Testing as Part of Personalized Medicine Interventions

**DOI:** 10.3389/fphar.2019.00830

**Published:** 2019-08-02

**Authors:** Stavros Simeonidis, Stefania Koutsilieri, Athanassios Vozikis, David N. Cooper, Christina Mitropoulou, George P. Patrinos

**Affiliations:** ^1^Department of Pharmacy, University of Patras School of Health Sciences, Patras, Greece; ^2^Economics Department, University of Piraeus, Piraeus, Greece; ^3^Institute of Medical Genetics, Cardiff University, Cardiff, United Kingdom; ^4^The Golden Helix Foundation, London, United Kingdom; ^5^Zayed Center of Health Sciences, United Arab Emirates University, Al-Ain, United Arab Emirates; ^6^Department of Pathology, College of Medicine and Health Sciences, United Arab Emirates University, Al-Ain, United Arab Emirates

**Keywords:** economic evaluation, pricing, reimbursement, genetic and genomic tests, personalized medicine, quality of life, willingness-to-pay

## Abstract

**Background:** The incorporation of genomic testing into clinical practice constitutes an opportunity to improve patients’ lives, as it makes possible the implementation of innovative, individualized clinical interventions that maximize efficacy and/or minimize the risk of adverse drug reactions. In order to ensure equal access to genomic testing for all patients, the costs associated with these tests should be reimbursed by their respective national healthcare systems. Given that funding for the public health sector is decreasing in real terms, it is of paramount importance that the emerging interventions are thoroughly evaluated both in terms of their clinical effectiveness and their full economic cost.

**Objective:** The aim of this study was to identify those genome-guided interventions that could be adopted and reimbursed by national healthcare systems. Further, we recorded the underlying factors determining the broad adoption of genome-guided interventions in clinical practice, in order to identify potential reimbursement criteria.

**Methods:** We performed a systematic review of published (PubMed-listed) scientific articles on the economic evaluation of those individualized clinical interventions that include genomic tests. Information on genomic tests reimbursed by the US Medicare program was also included. Subsequently, we correlated the regulatory guidance given for the interventions collated in our systematic review with the corresponding economic evaluation results and policies of the Medicare program. Regulatory guidance information was collected from the PharmGKB online knowledgebase and the Clinical Pharmacogenetics Implementation Consortium (CPIC).

**Results:** Most of the included studies constitute cost-utility analyses, in which the outcome of the interventions has been measured in quality-adjusted life years (QALYs) whereas an estimate of the total cost has been based upon direct medical cost data. Favorable economic evaluation results, as well as concrete evidence demonstrating the clinical utility of pre-emptive genotyping, are considered as prerequisites for the broad adoption and reimbursement of the costs incurred during genomic testing. Indicatively, pre-emptive HLA-B*5701 and *TPMT* testing before administration of abacavir and azathioprine, respectively, is reimbursed by Medicare based on both economic and efficacy evidence. Likewise, the medical necessary screening for *MMR* and *BRCA1/2* genes are reimbursed for high-risk populations.

**Conclusions:** Our findings further underline the need for further cost-utility analyses within different national healthcare systems, in order to promote the reimbursement of the cost of innovative genome-guided therapeutic interventions.

## Introduction

Genomic analysis constitutes the basic tool of personalized medicine, as it allows the identification of specific nucleotide changes in patient genomes, thereby delineating their variomes in relation to predisposition to genetic diseases and/or to the effectiveness or otherwise of specific therapeutic drugs or the likelihood of adverse drug reactions (Phillips et al., 2013; Lee et al., 2014; Phillips et al., 2016). It is thus reasonable to expect that the introduction of genomic testing in clinical practice will contribute to the rationalization of established drug-prescription regimens and lead to the design of new, individualized interventions with maximized efficacy and minimized adverse drug reactions (Phillips et al., 2013; Sun et al., 2013).

Health economics and economic evaluation together aim to allocate the limited resources available in the most effective ways in various healthcare systems (Williams, 1987). Faced with the challenge of achieving optimal benefit for patients, while maintaining the sustainability of national healthcare systems, economic evaluation analyses are deemed to be an essential part of the decision-making process as to whether a new intervention should or should not be adopted (Jonsson, 2009; McFarland, 2014). Further, it is of great importance both in terms of patients’ need and equal access that these innovative interventions are reimbursed by national healthcare systems. To this end, there is a need for scientists to provide health policymakers with evidence of clinical validity, utility data associated with the genomic tests, as well as reliable evidence of economic benefit (Snyder et al., 2014). In other words, it is essential to demonstrate i) the clinical utility of all pharmacogenomic biomarkers used and ii) support from reliable economic data demonstrating that reimbursing the cost of such genomic tests will not only improve patient life quality but also reduce the costs of the overall national healthcare expenditure while increasing the efficiency of the public healthcare sector by guiding patients to personalized treatment recommendations (Vozikis et al., 2016). However, the available clinical and financial data are still very limited, and as such, more reliable economic evaluation studies are urgently required (Snyder et al., 2014).

Most economic evaluation studies deal with cohort studies, be they prospective or retrospective, where a group of patients is monitored over time with respect to their progression in relation to a particular disease or after exposure to a given drug or risk factor (dos Santos Silva, 1999; Song and Chung, 2011). In economic evaluation studies, most of the prospective cohort studies involve hypothetical cohorts, based on hypothetical/simulated patients. In such cases, the characteristics of hypothetical patients correspond to the characteristics of real patients taken from the literature or previous clinical trials (*46*)^1^. Moreover, primary data pertaining to treatment efficacy and the clinical progression of patients are computer-simulated. Based on these data, scientists can follow hypothetical cohorts of patients over time (*77*, *79*).

There are four types of economic evaluation study, depending upon the way in which the outcome is measured and evaluated, namely, cost-minimization analysis (CMA), cost-effectiveness analysis (CEA), cost-utility analysis (CUA), and cost-benefit analysis (CBA) (Veenstra et al., 2000; McFarland, 2014), where the outcomes are measured in monetary units, number of life years gained (LYs), or quality-adjusted life years (QALYs), respectively. As a result, different utility values may be measured for a specific health state (Prieto and Sacristán, 2003). CUA studies are considered to be of the utmost importance (Veenstra et al., 2000), although they are subject to significant constraints. Common limitations include difficulties in expressing test utility and in interpreting test results, given the lack of actual clinical utility data and the heterogeneity of patients’ overall clinical features. As long as these limitations remain to be overcome, genomic tests are unlikely to be reimbursed from the public purse (Snyder et al., 2014). In spite of this, it is encouraging that the methodology of economic evaluation has improved considerably in recent years; at the same time, several solutions have been proposed in order to overcome the aforementioned constraints, such as conduct of sensitivity analysis and/or value of information analysis (Buchanan et al., 2013).

In economic evaluation analysis, cost is invariably monetized and is classified either as a direct or indirect medical cost, which is the cost associated with providing healthcare in order to deal with an illness or the cost related to the expenditure incurred by the patient and their family as a consequence of healthcare provision, respectively (Riewpaiboon, 2014). The cost of genomic testing falls into the category of direct medical costs and is therefore always taken into account by health economists in such economic evaluation studies. The so-called societal cost comprises both the direct non-medical cost and the indirect cost, for which there are no official records, and which may help to explain why societal cost is never reimbursed from public funds. If measurement of the total cost of an intervention is based solely on direct medical cost data, the analysis is from the health-payer perspective. By contrast, if both the direct medical cost and the societal cost are considered, the analysis can be said to be from a societal perspective (Russell et al., 1999).

In most cases, the time period of disease progression exceeds the period of economic evaluation; thus, there is need for measurement of future costs and outcomes resulting from the application of the new intervention, which is accomplished using a variety of models such as decision trees and Markov models for short-term and long-term analysis, relevantly (Naimark et al., 1997).

If an innovative intervention (in this case, genome-guided) outweighs the conventional intervention on the basis of scientific effectiveness data, the decision as whether or not to adopt it depends on its overall cost. If the total estimated cost (including the economic benefits of reducing the incidence of the disease) is lower than that of the conventional intervention, the new intervention may be described as “cost-saving” (or dominant) and its adoption by the national healthcare system is highly recommended. By contrast, the new intervention is said to be “dominated” by the conventional intervention as long as the former is more costly and less effective; in this case, the new intervention should be overruled or put on hold. More frequently, inclusion of genomic testing in clinical practice leads to novel interventions that exceed the cost of their predecessors but also prevail in terms of overall effectiveness. For this reason, it is important that economic evaluation analyses are performed in order for society to decide whether the extra cost is “worth” paying (in order to reap the societal benefits of genomic testing). The decision to adopt a new intervention depends on the amount of money that society is willing to spend on each QALY gained (willingness-to-pay threshold). Whether the incremental cost-utility ratio (ICUR) of an intervention lies below or above this threshold determines the likelihood (or not) of being adopted by the national healthcare system (Bertram et al., 2016).

The willingness-to-pay threshold varies with respect to the country (e.g., $50,000–$100,000/QALY in the United States, £20,000–£30,000/QALY in the UK, etc.). (Stolk et al., 2004; Mccabe et al., 2008). In the absence of officially declared thresholds, health economists suggest a range of thresholds based on the medical literature and other official data, such as the per capita gross domestic product as suggested by the WHO (Marseille et al., 2014).

The Medicare program is the largest third-party payer that provides reimbursement for medical services on behalf of US citizens (De Lew, 2000). Eligible for Medicare coverage are US citizens aged 65 or older who have worked in the US and have paid payroll taxes, employees in government agencies, as well as people under the age of 65 with certified disability. Insurance coverage can also be provided through the spouse’s work. The federal agency responsible for managing the Medicare program is the Centers for Medicare and Medicaid Services (CMS) and is responsible for concluding contracts with private insurance companies to serve as fiscal agents between healthcare service providers and the US government (De Lew, 2000).

Although genomic tests constitute a useful tool for the diagnosis and personalized treatment of genetic diseases, concerns about their validity and cost hinder their extensive use in clinical practice and, subsequently, reimbursement. Here, we have conducted a systematic review of scientific articles describing the economic evaluation of individualized interventions, with the aim of recording the important characteristics of economic evaluation analysis, as well as evaluating the different genomic tests in terms of both their cost and effectiveness. The main objective of this approach is to identify those interventions that are more likely to be reimbursed by national healthcare systems. Furthermore, we triangulated the regulatory guidance of the genome-guided interventions recorded in our study with the corresponding economic evaluation results and reimbursement policies of major programs, such as Medicare. Such a correlation should reveal the criteria that need to be met in order for a new therapeutic intervention to be broadly implemented in the clinic and then reimbursed.

## Methods

**Figure 1:**
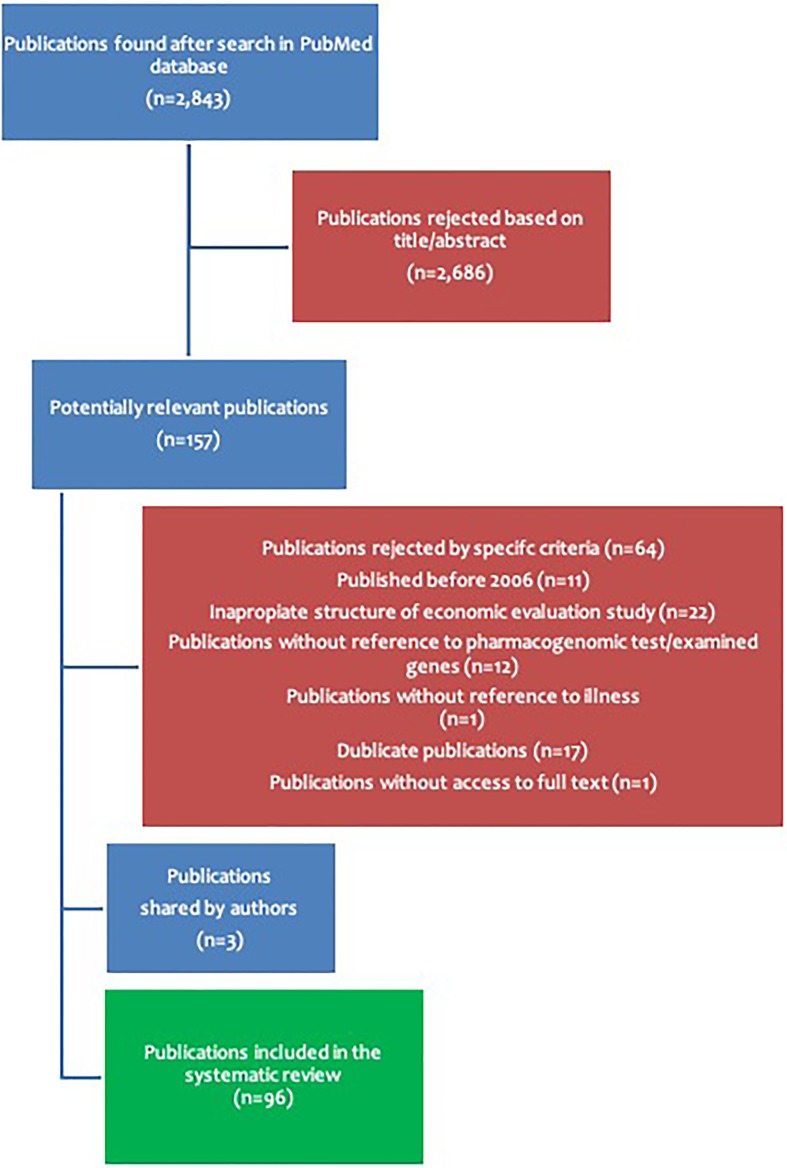
Overview of the article querying methodology in the PubMed literature database.

A systematic literature search was conducted using the PubMed database using the following search terms: “pricing and genomics,” “pricing and personalized medicine,” and “reimbursement and genomic tests.” Using the aforementioned terms, a total of 2,843 publications were collected, from which 2,686 publications were not processed further, based on their title and/or their abstract. From the remaining 157 publications, the full text was obtained in order to assess their suitability in accordance with the main purpose of this review, based on the following criteria. Firstly, only publications that were published since 2006 were included. Secondly, every enquiry had to be an economic evaluation of a therapeutic strategy involving one or more genomic tests assessed in comparison to another more or less applied therapeutic strategy not involving genomic tests. Another essential inclusion criterion was the nature of the economic evaluation study, which had to have been appropriately conducted. Moreover, clear reference must have been made either to the trade name of the test or the gene(s) examined, as well as to the disease(s) for which the therapeutic recommendations were designed. Based on the above criteria, 64 publications were rejected, leaving 96 publications to be further evaluated in this systematic review (**Supplementary Table 1**). The overall search methodology is depicted in **Figure 1**.

The 96 scientific articles were examined in detail in order to gather all the available information relevant to the scope of the present study. More specifically, we collected information pertaining to the nature of the economic evaluation studies (**Supplementary Table 2**) and the corresponding individualized interventions (**Supplementary Table 3**), while collating quantitative data, such as the cost of the genomic tests, the overall cost of the new interventions and their corresponding cost-utility, and/or cost-effectiveness ratios (**Supplementary Table 4**). Our literature mining effort was enriched with further data on those genomic tests that are reimbursed by the Medicare program. Emphasis was placed on the Medicare program, as its reimbursement policies constitute basic principles for many private insurance payers (Ball, 1995).

We subsequently cross-correlated the genome-guided therapeutic interventions reported in our literature review with the corresponding guidance from the American regulatory body, the Food and Drug Administration (FDA), as well as the online Clinical Pharmacogenetics Implementation Consortium (CPIC, 2019) resource (https://cpicpgx.org). Of note, we collected the FDA-approved drug labels *via* the PharmGKB knowledgebase (PharmGKB, 2019). “PharmGKB” is an online knowledge resource that integrates the pharmacogenomic information which is documented in the drug labels of major regulatory bodies, such as the FDA. In terms of the requirement to conduct a pharmacogenomic test before the administration of a given drug, pharmacogenomic testing may be classified as “required,” “recommended,” “actionable PGx,” or “informative PGx” in order of decreasing necessity (https://www.pharmgkb.org/). As far as the CPIC^®^ is concerned, it is an international consortium that provides freely available, evidence-based, and detailed gene/drug clinical practice guidelines in an effort to overcome the barrier of the implementation of pharmacogenomic testing in the routine clinical setting. CPIC has already published 21 guidelines covering 44 gene-drug pairs that have been meticulously curated and systematically updated. For each gene-drug pair, CPIC assigns a corresponding level ranging from A–D (in order of decreasing necessity). As far as the gene-drug correlations classified as CPIC level A are concerned, it is highly recommended that prescription of the affected drug should be changed with regard to the available genetic information, given that the strength of such a recommendation is of high or moderate importance. CPIC level B supports similar action in terms of clinical context while bearing in mind that alternative therapies/dosing are likely to be as effective and as safe as non-genetically based dosing (https://cpicpgx.org/).

## Results

**Figure 2:**
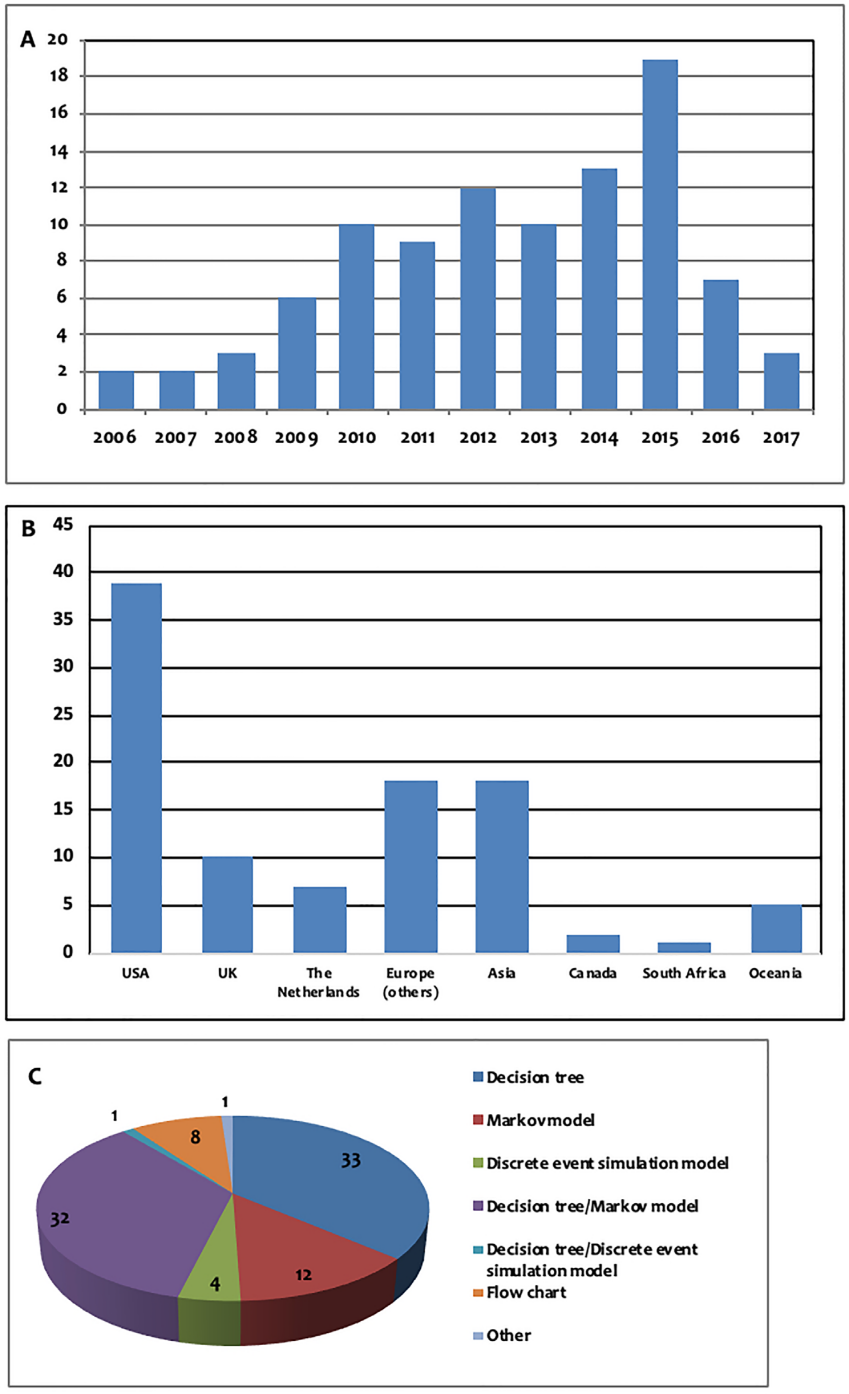
**(A)** Number of publications per year, **(B)**: number of publications per continent/country: Europe (others): Austria (*n* = 1), France (*n* = 2), Germany (*n* = 2), Denmark (*n* = 1), Switzerland (*n* = 3), Sweden (*n* = 1), Spain (*n* = 5), Italy (*n* = 1), Croatia (*n* = 1), Serbia (*n* = 1), Asia: China (*n* = 5), Japan (*n* = 3), South Korea (*n* = 1), Singapore (*n* = 4), Thailand (*n* = 3), Oceania: Australia (*n* = 3), New Zealand (*n =* 2), **(C)**: models used for measurement of future costs and outcomes.

In the present systematic review, we examined only economic evaluation studies published since 2006, the majority of which were published after 2010. More specifically, only 23 publications were published between 2006 and 2010, while 73 publications were published between 2010 and the present. The number of studies published per year is presented in **Figure 2A**. Moreover, the majority of the studies were performed either in a European country (34 studies) or in the USA (41 studies). The majority of the economic evaluation studies were conducted in two countries, namely, the USA (39 studies) and the United Kingdom (10 studies). The number of publications per country is presented in **Figure 2B**.

### Qualitative Characteristics of Economic Evaluation Studies

#### Cohort Study and Economic Evaluation Model

In this systematic review, 83 prospective cohort studies and 13 retrospective cohort studies were identified. It should be noted that 80 of the 83 prospective studies were based on hypothetical cohorts of patients, while only three were based on real patients. The models used with regard to the measurement of future costs and outcomes are presented in **Figure 2C**.

#### Measurement of Outcome and Cost

In our systematic review, 27 studies employed CUA, 17 CEA, while only two adopted CMA. 40 publications employed a combination of CEA and CUA, whereas one was a combination of CEA and CMA. The remaining nine publications were simple economic analyses, as the interventions were evaluated taking into consideration only the total cost of each intervention.

**Figure 3:**
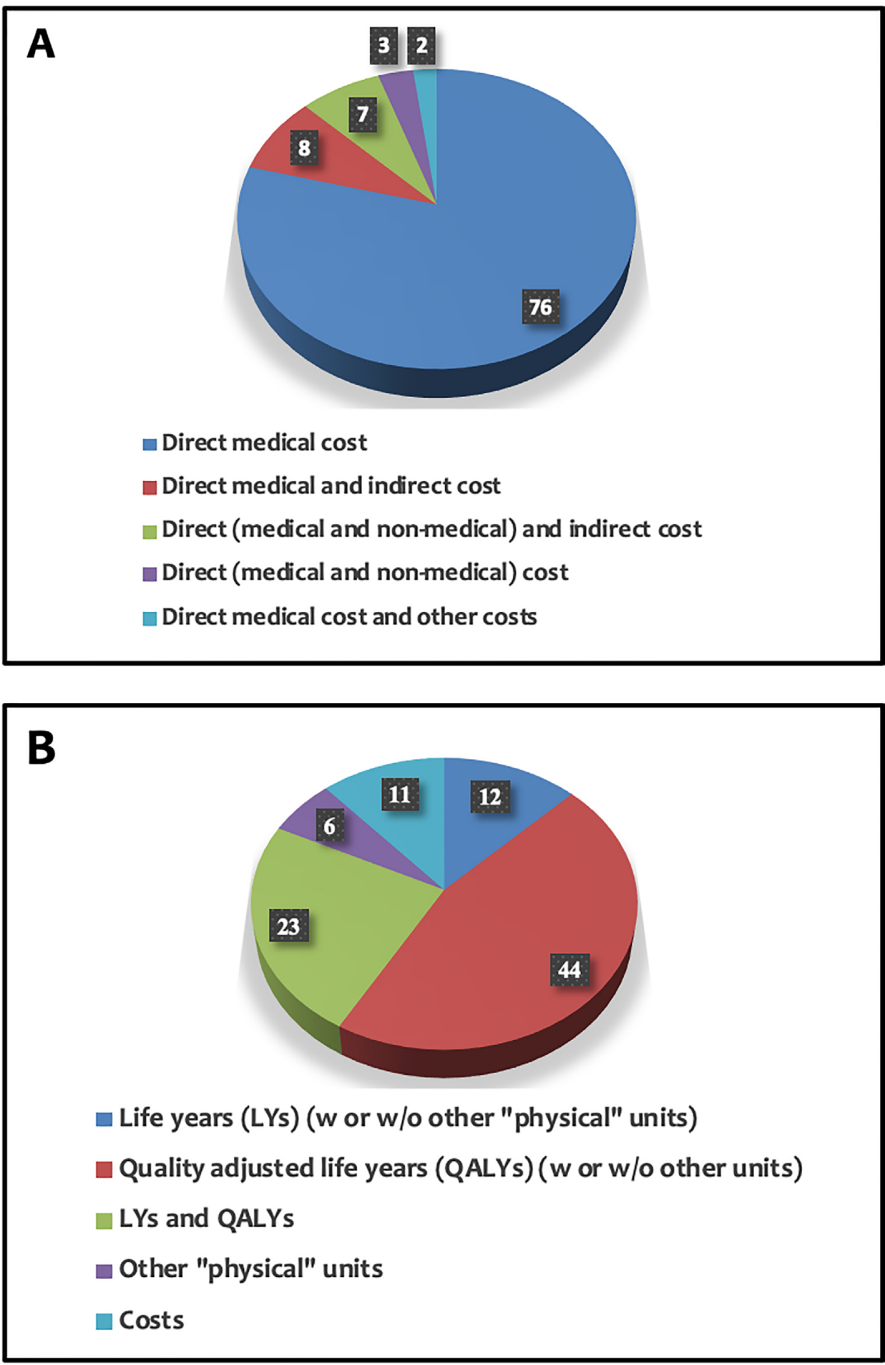
Measurement of cost **(A)** and outcome **(B)** in the articles analyzed within this study.

Further, regarding the measurement of the outcome, in 44 publications, the outcome was measured in QALYs and in 12 publications in LYs, whereas in 23 publications, a combination of QALYs and LYs was preferred. In the vast majority of the publications included in this study (76 out of 96 studies), the researchers took into consideration only the direct medical cost when determining the total cost of individualized interventions. The applied methods of measuring the outcome and cost in all studies are presented in **Figure 3**.

### Estimation of Cost-Effectiveness and Cost-Utility of Individualized Interventions

In our systematic review, we attempted to establish which interventions were likely to be adopted by each national healthcare system in their corresponding countries, based on the available willingness to pay threshold. The “cost-saving” interventions suggested to be adopted and reimbursed are shown in **Table 1**. Moreover, the interventions that were found to be either cost-effective or not, according to ICUR data, and the willingness to pay thresholds (suggested by authors) are presented in **Supplementary Tables 5A** and **Supplementary 5B**, respectively.

**Table 1:** Cost and number of quality-adjusted life years (QALYs) of “dominant” individualized interventions.

Disease	Gene	Cost of intervention		Number of QALYS		Reference^++^
		W/o PGx test/test 1	With PGx test/test 2	W/o PGx test/test 1	With PGx test/test 2	
**Advanced adenocarcinoma** **of the lung**	*EGFR*	SG$47,100	SG$44,700	0.87	0.91	(Lee et al., 2014)
**Colorectal cancer**	*KRAS*	¥3,160,000($35,000)	¥2,600,000 ($29,000)	0.48	0.49	(Sun et al., 2013)
	*UGT1A1*	$13,058	$12,786	1.6347	1.6349	(*54*)
**Acute coronary syndrome**	*CYP2C19*	$15,800	$14,900	0.966	0.9665	(Jonsson, 2009)
		NZ$85,342	NZ$84,646	8.544	8.650	(Hess et al., 2015)
		$76,906* (CLO)	$76,450	7.4381	7.5301	(*47*)
		$78,296 (P2Y12)				
**Neonatal diabetes**	*KCNJ11, ABCC8*	$71,784	$59,256	16.29	16.99	(Song and Chung, 2011)
**Atrial fibrillation**	*4q25*	Cost saving of $250,689		Net gain of 8.8		(Riewpaiboon, 2014)
**Familial adenomatous polyposis**	*APC*	€13,928.82 (Spain)	€8,038.93	19.92	19.93	(*65*)
**Neovascular macular degeneration**	*CFH, ARMS2/HTRA1, C3, C2, CFB*	Cost saving of $493		Gain of 0.0392		(*78*)
**Venous thromboembolism**	Thrombo inCode^®^	€1,366.30–€2,795.61 (Spain)	€832.58–€848.38	8.2586–8.4784	8.5871–8.5874	(*61*)
**Breast cancer**	MammaPrint^®^	€17,869^+^ (Spain)	€16,989	18.131	18.357	(Snyder et al., 2014)
		$27,882^+^	$21,598	7.364	7.461	(*53*)

*Currency used is different from the currency of the country in which the corresponding economic evaluation study was carried out. Researchers chose to express costs in US $ ($). ^+^Cost of individualized intervention including Oncotype DX^®^. PRA, prasugrel; CLO, clopidogrel; P2Y12, other P2Y12 inhibitor (individualized interventions including prasugrel, clopidogrel and other P2Y12 inhibitors, respectively). ++Identification number of the article (**Supplementary Table 1**).

### Regulatory Instructions and Economic Evaluation in Reimbursement Decisions

Subsequently, we aimed to cluster the pharmacogenomic correlations for which we have collected evidence from the Medicare program regarding the corresponding guidance and economic studies conducted (**Tables 2** and **3**). As may be readily assumed, those gene-drug correlations that have the strongest evidence to support the necessity of genetic testing, and that are accompanied by reliable cost-utility studies which prove the cost-effectiveness of pre-emptive genotyping, are those that are reimbursed. Abacavir-HLA-B*5701 probably constitutes the best example of the concordance between scientific and economic data. Indeed, both the FDA and CPIC highlight the contraindication of this medication to carriers of the HLA-B*5701 allele due to the high risk of hypersensitivity reactions (HSR) (Centers for Medicare & Medicaid Services, 2018; BlueCross Blueshield of Western New York, 2018; Quest Diagnostics, 2015; PGX Tests Determined to be Medicially Necessary for Medicare Coverage.; Local Coverage Determination (LCD)), while economic evaluation results with an ICUR of $36,700/QALY (lower than the $50,000/QALY threshold) justify its cost-effectiveness (*60*). Another noteworthy gene-drug correlation is the *TPMT*-azathioprine; pre-emptive genotyping (for the administration of this drug) is classified as level A by CPIC and “recommended” by the FDA, indicating that changes in dosing should be done with regard to the pharmacogenomic results. Apart from the aforementioned strong clinical evidence, adoption and reimbursement of *TPMT* testing (Local Coverage Determination (LCD); Blue Regence, 2019) are also supported by the results of economic evaluation, given that *TPMT* testing in idiopathic pulmonary fibrosis prior to azathioprine treatment has an ICUR of $49,156/QALY, below the US $50,000/QALY threshold (*27*).

**Table 2:** Pharmacogenomic tests covered by Medicare.

Drug	Allele	FDA	CPIC	ICUR	Willingness-to-pay threshold	Type	Reference^++^
**Abacavir**	HLA-B*5701	Required	A	$36,700/QALY	$50,000–$100,000/QALY	CE	(*60*)
				–	–	DT	(*64*)
**Azathioprine**	*TPMT*	Recommended	A	$49,156/QALY	$50,000/QALY	CE	(BlueCross Blueshield of Western New York, 2018)
**Carbamazepine**	HLA-B*1502 *^1^*	Required	A	$85,697/QALY	$50,000/QALY	Not CE	(Stolk et al., 2004)
				$29,750/QALY	$50,000/QALY	CE	(De Lew, 2000)
**Cetuximab**	*KRAS*	Required	–	–	–	Cost saving, same effectiveness	(*44*)
**Clopidogrel**	*CYP2C19* *^2^*	Actionable	A	–	–	Dominant	(Jonsson, 2009, *47*)
				$4,200/QALY	$100,000/QALY	CE	(Meckley and Neumann, 2010)
**Crizotinib**	*ALK*	Required	–	$136,000/QALY	$200,000/QALY	CE	(Blue Regence, 2019)
**Erlotinib**	*EGFR*	Required	–				
**Erlotinib**	*EGFR*	Required	–	$110,658/QALY	$100,000/QALY	Not CE	(*86*)
				$162,018/QALY	$150,000/QALY	Not CE	(*63*)
**Panitumumab**	*KRAS*	Required	–	–	–	Cost saving, same effectiveness	(*44*)
**Phenytoin**	HLA-B*1502 *^1^*	Actionable	A	$85,697/QALY	$50,000/QALY	Not CE	(Stolk et al., 2004)
				$29,750/QALY	$50,000/QALY	CE	(De Lew, 2000)
**Trastuzumab**	*ERBB2 (HER-2)*	Required	–	–	–		
**Vemurafenib**	*BRAF*	Required	–	–	–		

^1^HLA-B*1502 is regarded medically necessary and thus reimbursed only for patients of Asian ancestry. ^2^CYP2C19 testing is regarded as medically necessary only for patients with ACS undergoing PCI who are initiating or reinitiating Clopidogrel therapy. CE, Cost-effective; DT, Dominant. ^++^Identification number of the article (**Supplementary Table 1**).

**Table 3:** Genomic tests covered by Medicare.

Allele	FDA	CPIC	ICUR	Willingness-to-pay threshold	Type	Reference^++^
**MMRgenes**	–	–	$26,000/QALY	$50,000/QALY	CE	(*91*)
***BRCA1/2*** **^3^**	–	–	$9,000/QALY	$50,000/QALY	CE	(Gavan et al., 2018)

^3^BRCA1/2 testing is regarded as medically necessary only for patients with breast cancer and healthy individuals with a family history of breast cancer. ^++^Identification number of the article (**Supplementary Table 1**).

In our literature mining effort, we also identified pharmacogenomic correlations with variable economic results. Regarding the cost-effectiveness of HLA-B*1502 genotyping in relation to carbamazepine or phenytoin treatment, the results differ; some of them demonstrate that pharmacogenomic testing is cost-effective, whereas others do not. However, it is noteworthy that carbamazepine has a boxed warning on its FDA drug label stating that screening for HLA-B*1502 allele is required to be carried out prior to treatment in patients that are genetically at-risk due to the high risk of serious and sometimes fatal dermatological reactions. CPIC guidance concurs with that of the FDA, strongly recommending the use of an alternative drug in case patients are HLA-B*1502 carriers and carbamazepine-naïve. Similarly, both on the FDA phenytoin label and in the corresponding CPIC guideline, it is documented that consideration should be given to avoiding phenytoin as an alternative for carbamazepine in patients positive for HLA-B*1502. Therefore, even though the economic results cannot lead to a definite conclusion as far as the reimbursement policy that should be applied, the clinical evidence that lies behind these pharmacogenomic correlations supports the broad clinical adoption of pre-emptive testing in patients of Asian ancestry (BlueCross Blueshield of Western New York, 2018; IGNITE Implementing GeNomics In practice, 2018; Local Coverage Determination (LCD)).

With the exception of those gene-drug correlations whose economic data are inconclusive, there are also pharmacogenomic biomarkers whose economic benefits remain to be assessed or whose economic results clearly discourage reimbursement. According to the FDA drug label, erlotinib is a tyrosine kinase inhibitor indicated for first-line treatment of patients with metastatic non-small cell lung cancer (NSCLC), whose tumors are characterized by epidermal growth factor receptor (*EGFR*) exon 19 deletions or exon 21 (p.L858R) missense mutations. The FDA indicates that patients being considered for erlotinib treatment should first be tested for the aforementioned mutations by means of an FDA-approved test as neither the safety nor the efficacy of erlotinib have been established in NSCLC patients whose tumors have other *EGFR* mutations. *EGFR* testing is part of the Medicare reimbursement fee schedule (Centers for Medicare & Medicaid Services, 2018; PGX Tests Determined to be Medically Necessary for Medicare Coverage; Local Coverage Determination (LCD); Hess et al., 2015), despite the fact that no economic evaluation analysis has been performed which shows that testing for over-expression of *EGFR* prior to erlotinib treatment can be cost-effective; in all analyses, the new interventions exceed the $100,000/QALY threshold (*63*, *86*). However, the decision to allow reimbursement appears to have been influenced by strong clinical evidence documented on the FDA drug label, suggesting that testing for overexpression of EGFR contributes to achieving an optimal therapeutic effect in both lung and colon cancers. The example of vemurafenib-*BRAF* should also be mentioned, as this pharmacogenomic test is also reimbursed by Medicare (Centers for Medicare & Medicaid Services, 2018; IGNITE Implementing GeNomics In practice, 2018; Local Coverage Determination (LCD); Hess et al., 2015) even in the absence of economic evaluation studies. Based on the mechanism of action of this tyrosine kinase inhibitor, the FDA drug label states that vemurafenib is only indicated for the treatment of patients with unresectable or metastatic melanoma with *BRAF* p.V600E mutation as detected by an FDA-approved test.

As far as genomic tests are concerned, we aimed to cross-correlate the Medicare reimbursement policies with results from cost-utility analyses from our systematic review. In general, Medicare covers genomic tests that are regarded as medically necessary by the Centers of Medicare & Medicaid Services. More specifically, screening for *MMR* variants in colorectal tumors is regarded as medically necessary only for colorectal cancer patients (and then only for whose family members meet specific criteria/the revised Bethesda guidelines) (Local Coverage Determination (LCD)). Precautionary *MMR* testing in individuals who are at-risk of developing colorectal cancer and/or Lynch syndrome is considered experimental and hence not medically necessary. It is encouraging though that there are economic evaluation results which suggest that precautionary testing for *MMR* mutations in unaffected individuals with a family history of colorectal cancer is cost-effective, with an ICUR of $26,000/QALY (*91*). Another example is testing for *BRCA1/2* genes, which constitute well-established pharmacogenomic biomarkers for breast cancer, as specific mutations in these genes have been associated with a greatly increased risk of developing breast cancer. *BRCA1/2* testing is covered mostly for affected individuals with a family history of breast cancer and occasionally for healthy individuals with suspected breast cancer and/or breast cancer history (Centers for Medicare & Medicaid Services, 2018; Local Coverage Determination (LCD); Beattie et al., 2012; Meckley and Neumann, 2010). However, economic evaluation results indicate that precautionary testing for *BRCA1/2* is cost-effective with an ICUR of $9,000/QALY) even in healthy/unaffected women with a family risk of breast cancer (*36*).

## Discussion

Personalized medicine targets health care interventions to subgroups of patients, who share specific biological and genetic characteristics. The most commonly used applications are genomic tests, which dominate the era of personalized medicine and, thus, constitute the main focus of this study, as far as their pricing and reimbursement are concerned. Pre-emptive genotyping leads to new individualized drug treatment interventions, where the appropriate drug is administered to each patient in an effort to minimize the incidence of drug toxicity or lack of efficacy. Hence, diseases are treated more effectively, while the quality of the patient’s life improves. At the same time, national healthcare systems benefit from the expected reduction in expenditure on unnecessary medical procedures and/or the hospitalization of patients suffering from adverse drug reactions resulting from inadequate therapies. However, it should be noted that personalized medicine also includes other forms of applications, such as algorithm-based prescribing, population-based screening programs etc., which were not taken into consideration in the present study (Gavan et al., 2018; Vizirianakis et al., 2019).

The heterogeneity of patients’ specific characteristics (phenotype) due to genome-variants leads to the heterogeneity in patients’ drug treatment response and/or development of adverse drug reactions. Researchers consider this patient-level heterogeneity, while conducting economic evaluation analysis, as it affects both total treatment costs and outcomes (Gavan et al., 2018). This is why many national health care agencies, for instance the National Institute for Health and Care Excellence (NICE) for England and Wales, suggest that sub-group analyses should be conducted in order to make decisions about implementation of new health technologies (including genomic tests) in clinical practice (Espinoza et al., 2014).

Genomic tests are also directed to particular populations, for example, patients suffering from rare diseases that cannot be easily treated with conventional interventions. Indicatively, in our systematic analysis, there were studies, in which pre-emptive genotyping for the diagnosis of rare diseases (hypertrophic cardiomyopathy, Cowden syndrome, neovascular macular degeneration, neurofibromatosis etc.) was evaluated. Moreover, there are applications of genomic tests even in unborn children. In our systematic review, we identified studies evaluating prenatal screening for spinal muscular atrophy, cystic fibrosis, and X-linked hemophilia.

Two essential parameters emerge as fundamental preconditions for a pharmacogenomic test to be broadly adopted in the clinic and for it to be reimbursed: concrete evidence of the relevant gene-drug correlation, as well as favorable economic evaluation results. As implied by our literature review, the strong classification of the pharmacogenomic information by the CPIC and/or FDA guidance constitutes a key factor in reimbursement decision making, even in the absence of favorable economic results as illustrated by the pharmacogenomic correlations of vemurafenib-*BRAF* and erlotinib-*EGFR*. Pre-emptive genotyping is often deemed crucial for a specific population, the so-called high-risk groups. In particular, treatment with carbamazepine is strongly associated with a high risk of developing Stevens-Johnson syndrome and toxic epidermal necrolysis (SJS/TEN) in carriers of the HLA-B*1502 allele. Given that this inherited allelic variant is mainly observed in patients of Asian ancestry, the corresponding pharmacogenomic test is only reimbursed for patients with Asian ancestry (Centers for Medicare & Medicaid Services, 2018; Local Coverage Determination (LCD)). In parallel, although *BRCA1/2* constitutes a well-established genomic biomarker for the development of breast cancer, *BRCA1/2* genetic testing is considered medically necessary and, thus, uniquely covered for individuals with a family history of such a disease (Beattie et al., 2012).

As far as the economic evaluation results are concerned, it is of paramount importance that the pharmacogenomic tests are proven to be either “cost-saving” or at least cost-effective in economic terms, in other words, that the proposed therapeutic intervention is also cost-saving, apart from being more effective, than the already established one. Given that the inclusion of pre-emptive genotyping often increases the cost of the therapeutic recommendation, the eventual decision depends upon the willingness-to-pay thresholds, which serve to ensure the affordability of the new interventions. An indicative example of just such a cost-effective pharmacogenomic test which meets the aforementioned criteria is HLA-B*5701 genotyping prior to the initiation of abacavir treatment in HIV patients. More specifically, the agreement of both FDA and CPIC about the necessity of pre-emptive HLA-B*5701 genotyping, as well as the favorable economic evaluation results ($36,700/QALY at a willingness-to-pay threshold of $50,000/QALY), justifies the reimbursement of this pharmacogenomic test by Medicare (*60*).

In our study, we focused on the US Medicare program, as there are limited data regarding reimbursement of genomic testing in countries of the European or Asian region. More specifically, as far as the European Union is concerned, each member state has a different reimbursement policy, as each country spends a different amount of budget on the health sector. In addition, there is a different percentage of private and public insurance contribution in reimbursement of healthcare services. Some countries have approved reimbursement exclusively from public or private insurance funds, while others from a combination of them. As a result, there is no uniform regulatory framework providing precise instructions and provisions on the conditions and the exact procedure for reimbursement of genomic tests from the public funds (Vozikis et al., 2016).

Most economic evaluation studies in our systematic review were cost-utility analyses. However, the credibility of economic evaluation analysis is negatively affected by the lack of actual clinical utility data from genomic testing in real patients (Snyder et al., 2014). Indeed, 80 out of 96 studies in our systematic review were based on hypothetical cohorts, where hypothetical patients and simulated clinical data from older clinical trials were used. Furthermore, the use of retrospective cohorts also raises concerns about the quality of the results produced. There were 13 retrospective studies in our systematic review, which accounts for a significant proportion of the total number of publications. More specifically, a retrospective study design may be associated with poorer data quality, as it is based on data from healthcare databases that have already been collected. This increases the risk of selection bias, which refers to the selection of inappropriate individuals that are unrepresentative of the population that researchers wish to study. Moreover, inaccurate or incomplete recollections from the past of the cohort’s individuals (recall bias) may also lead to questionable economic evaluation results.

Elaborating more on the economic evaluation method, economic evaluation from a societal perspective is considered more complete and more reliable, in comparison to the corresponding analysis from the health-payer perspective (Jonsson, 2009). It is well known that due to the lack of official societal cost recordings and the general targeting of reimbursement programs in the so-called direct medical cost, most studies are orientated from the health-payer perspective. This tendency, which is also confirmed by our systematic review results, indicates the need for more cost-utility analyses from the societal perspective, in order to allow optimal (societal) decisions to be made. It is encouraging that the methodology of economic evaluation has improved significantly with the development of statistical models that enable forecast of the interventions’ overall cost and clinical effectiveness over time, providing even life-time analysis (Naimark et al., 1997; Payne et al., 2018). More specifically, in 31 studies covered in our systematic review, both short-term and long-term analyses were achieved using a combination of decision trees and Markov models. Taking into account 11 additional studies in which only Markov models were used, long-term analysis was achieved in almost half of the studies under this systematic review.

In model-based economic evaluation analyses, uncertainty may also arise because of difficulties in estimating the true value of varying parameters (variables), which are used in the aforementioned models. Such variables usually constitute the cost of health care interventions or the age of patients. The models used in economic evaluation offer the opportunity to estimate the impact of parameter uncertainty using probabilistic sensitivity analysis. Taking into consideration the potentiality of long-term analysis of incremental costs and outcomes, it is of no doubt that model-based economic evaluation constitutes the preferred approach in decision making (Payne et al., 2018).

### Conclusions and Future Perspectives

National healthcare systems are often unable to cope with the ever-increasing social needs for high-quality healthcare service provision. Over the decades, the rapid growth of the population, the increasing economic resources of national economies, and the increasing cost of healthcare provision have led to a marked increase in the annual health expenditure in Western countries (McFarland, 2014). However, funding for the public health sector has decreased since the 2008 financial crisis, and as a result, qualitative selection among different medical interventions has to be made.

In relation to the role of economic evaluation in public health policy-making, there is an urgent need for the establishment of national policies that favor the conduct of economic evaluation in state-owned research institutes and universities, as well as in the private sector. It is encouraging that in recent years, the number of published economic evaluation analyses in the field of genomic and personalized medicine has continued to increase. This tendency accords with our systematic review results, which highlight the increasing number of publications since 2011. However, and in accordance with our findings from this study, more cost-utility analyses should be conducted in various countries in order to cover as many populations and ethnic groups as possible. By contrast, only a few relevant analyses have been conducted in Asian and African countries. Given the high frequency of high-risk and actionable alleles in Asian and African populations, it might reasonably be expected that researchers would be especially interested in economic evaluation of genomic testing in these countries. Taking into consideration the recorded mortality rates in low income Asian and African countries, pharmacogenomic research would contribute to the mitigation/prevention of global health inequalities.

Another crucial issue to be investigated is the economic thresholds. Based on our findings, there are no strictly defined willingness-to-pay thresholds even for a specific country’s national health system. Given existing social inequalities, health economists suggest that the commonly used thresholds should be expanded. The lack of concordance between the budgetary capability of the national health systems and the needs of local societies have led to the use of expanded willingness-to-pay thresholds suggested by health economists (Eichler et al., 2004; Neumann et al., 2014). In other words, it is likely that the persistence in strictly defined economic thresholds could lead to a fruitless controversy between public health providers and specific social groups or patients, while underestimating the scientifically proven clinical utility of genomic testing. Moreover, the interventions under evaluation are developed against diseases, which differ in terms of their severity, their pathophysiological mechanisms, and, consequently, their treatment regimens and cost. As a result, apart from social inequalities and other socio-economic factors, it would be scientifically inappropriate to use a strictly defined threshold for universal assessment of dissimilar interventions.

Many studies considered in this systematic review concluded that genomic-guided treatment may represent a cost-saving or cost-effective strategy against various diseases, including different types of cancer. Many of these strategies include genomic tests that are reimbursed by the US Medicare program, which is indicative of the leading role of economic evaluation results in determining reimbursement policymaking. Unsurprisingly, most of the non-cost-effective interventions are not covered by Medicare. Apart from the unfavorable economic evaluation results, which clearly do not provide a cogent argument for reimbursement, the decision not to cover the cost of the relevant genomic tests is mainly attributed to insufficient evidence supporting their clinical utility (Hess et al., 2015; Local Coverage Article: MolDX). It should be mentioned that this claim is more aligned with FDA regulations than with CPIC guidance.

Furthermore, our systematic review results emphasize the wide range of potential genomic testing applications, including interventions against colorectal and breast cancer, as well as acute coronary syndrome, cardiovascular disease, neonatal diabetes, and macular degeneration (**Table 1**, **Supplementary Table 5A**). Additional analyses could usefully be performed in order to enrich the already favorable economic evaluation data, in an effort to ensure positive reimbursement decisions by the national healthcare systems.

Last, but not least, the adoption of an appropriate universal legal framework is deemed necessary in order to determine the appropriate conditions for reimbursement of clinically valid tests. It should be noted that a basic precondition for achieving this goal is the foundation of a stable, effective, and transparent pricing system to avoid overpricing.

## Data Availability

All datasets generated for this study are included in the manuscript/supplementary files.

## Author Contributions

GP and CM conceived the study, AV, SS and SK conducted the analysis. DC, CM and GP commented on the results. All authors have written, read and approved the final manuscript.

## Funding

This study has been supported in part by a European Commission grant (U-PGx; H2020-668353) to GP and CM. SK is a recipient of an Onassis Foundation scholarship. The authors declare no conflict of interests. GP is full member and National Representative at the European Medicines Agency, CHMP-Pharmacogenomics Working Party.

## Conflict of Interest Statement

The authors declare that the research was conducted in the absence of any commercial or financial relationships that could be construed as a potential conflict of interest.

## Footnotes

1 The in-text references in italics represent the identification number of the article included in the systematic review literature as shown in **Supplementary Table 1**.
