# Supplementary material for: Application of Economic Evaluation to Assess Feasibility for Reimbursement of Genomic Testing as Part of Personalized Medicine Interventions
Source: Front Pharmacol. 2019 Aug 2;10:830. doi: 10.3389/fphar.2019.00830 (PMC6688623; doi:10.3389/fphar.2019.00830)
Supplement: Supplementary file 1 [file Table_1.docx]

**Supplementary Information**

**Application of economic evaluation to assess feasibility for reimbursement of genomic testing as part of personalized medicine interventions**

Stavros Simeonidis^1,#^, Stefania Koutsilieri^1,#^, Athanassios Vozikis^2^, David N. Cooper ^3^, Christina Mitropoulou^4^, George P. Patrinos^1,5,6^

^1^ University of Patras School of Health Sciences, Department of Pharmacy, Patras, Greece;

^2^ University of Piraeus, Economics Department, Piraeus, Greece;

^3^ Cardiff University, Institute of Medical Genetics, Cardiff, UK;

^4^ The Golden Helix Foundation, London, UK;

^5^ United Arab Emirates University, Zayed Center of Health Sciences, Al-Ain, UAE

^6^ United Arab Emirates University, College of Medicine and Health Sciences, Department of Pathology, Al-Ain, UAE;

^#^ These authors contributed equally to this work

| Supplementary Table 1. Literature used for the systematic review | | | | |
| --- | --- | --- | --- | --- |
| r/n | **Title** | **Authors** | **Journal** | **Publication year** |
| 1 | Cost-effectiveness analysis of alternative screening and treatment strategies for heterozygous familial hypercholesterolemia in the United States | Christina X. Chen, Joel W. Hay | International Journal of Cardiology | 2015 |
| 2 | Cost-Effectiveness of Epidermal Growth Factor Receptor Mutation Testing and First-Line Treatment With Gefitinib for Patients With Advanced Adenocarcinoma of the Lung | Gilberto de Lima Lopes Jr, Joel E. Segel, Daniel S.W. Tan, Young K. Do, Tony Mok, Eric A. Finkelstein | Cancer | 2012 |
| 3 | A Cost Effectiveness Analysis of Thiopurine Methyltransferase Testing for Guiding 6-Mercaptopurine Dosing in Children With Acute Lymphoblastic Leukemia | Jennifer R. Donnan, Wendy J. Ungar, Maria Mathews, Rebecca L. Hancock-Howard, Proton Rahman | Pediatric Blood &Cancer | 2011 |
| 4 | Cost-Effectiveness Analysis of KRAS Testing and Cetuximab as Last-Line Therapy for Colorectal Cancer | Takeru Shiroiwa, Yoshiharu Motoo, Kiichiro Tsutani | [Molecular Diagnosis & Therapy](https://link.springer.com/journal/40291) | 2010 |
| 5 | Cost effectiveness of high-risk HPV DNA testing for cervical cancer screening in South Africa | Arthi Vijayaraghavan, Molly Efrusy, Gerhard Lindeque, Greta Dreyer, Christopher Santas | Gynecologic Oncology | 2009 |
| 6 | Cost-effectiveness of newborn screening for cystic fibrosis determined with real-life data | C.P.B. van der Ploeg, M.E. van den Akker-van Marle, A.M.M. Vernooij-van Langen, L.H. Elvers, J.J.P. Gille, P.H. Verkerk, J.E. Dankert-Roelse | Journal of Cystic Fibrosis | 2014 |
| 7 | Genetic testing in patients with acute coronary syndrome undergoing percutaneous coronary intervention: a cost-effectiveness analysis | A. Lala, J.S. Berger, G. Sharma, J.S. Hochman, R. Scott Braithwal TE, J.A. Ladapo | Journal of Thrombosis and Haemostasis | **2013** |
| 8 | Genomic profile of breast cancer: cost–effectiveness analysis from the Spanish National Healthcare System perspective | Miguel Angel Seguı´, Carlos Crespo, Javier Corte', Ana Lluch, Max Brosa, Virginia Becerra, Sebastia'n Matias Chiavenna, Alfredo Gracia | Expert Review of Pharmacoeconomics & Outcomes Research | 2014 |
| 9 | Cost-Effectiveness of Genetic Testing in Family Members of Patients With Long-QT Syndrome | Marco V. Perez, Narmadan A. Kumarasamy, Douglas K. Owens, Paul J. Wang, Mark A. Hlatky | Circulation. Cardiovascular Quality and Outcomes | 2011 |
| 10 | Cost-Effectiveness Analysis of a Genetic Screening Program in the Close Relatives of Spanish Patients With Familial Hypercholesterolemia | Juan Oliva, Julio López-Bastida, Santiago G. Moreno, Pedro Mata, Rodrigo Alonso | Revista Española de Cardiología | 2009 |
| 11 | The Cost-Effectiveness of Personalized Genetic Medicine The case of genetic testing in neonatal diabetes | Siri Atma W. Greeley, Priya M. John, Aaron N. Winn, Joseph Ornelas, Rebecca B. Lipton, Louis H. Philipson, Graeme I. Bell, Elbert S. Huang | Diabetes Care | 2011 |
| 12 | DNA testing for hypertrophic cardiomyopathy: a cost-effectiveness model | Sarah Wordsworth, Jose´ Leal, Edward Blair, Rosa Legood, Kate Thomson, Anneke Seller, Jenny Taylor, Hugh Watkins | European Heart Journal | 2010 |
| 13 | Cost-effectiveness of a Genetic Test for Breast Cancer Risk | Henry J. Folse, Linda E. Green, Andrea Kress, Richard Allman, and Tuan A. Dinh | Cancer Prevention Research | 2013 |
| 14 | Cost-Effectiveness of UGT1A1*28 Genotyping in Preventing Severe Neutropenia Following FOLFIRI Therapy in Colorectal Cancer | Solen Pichereau, Anne Le Louarn, Thierry Lecomte, Hélène Blasco, Chantal Le Guellec and Hélène Bourgoin | Journal of Pharmacy & Pharmaceutical Sciences | 2010 |
| 15 | Genetic risk for atrial fibrillation could motivate patient adherence to warfarin therapy: a cost effectiveness analysis | Dov Shiffman, Marco V. Perez, Lance A. Bare, Judy Z. Louie, Andre R. Arellano and James J. Devlin | BMC Cardiovascular Disorders | 2015 |
| 16 | Societal cost-effectiveness analysis of the 21-gene assay in estrogen-receptor–positive, lymph-node–negative early-stage breast cancer in Japan | Hideko Yamauchi, Chizuko Nakagawa, Shinji Yamashige, Hiroyuki Takei, Hiroshi Yagata, Atsushi Yoshida, Naoki Hayashi, John Hornberger, Tiffany Yu, Calvin Chao, Carl Yoshizawa, Seigo Nakamura | BMC Health Services Research | 2014 |
| 17 | A five-year model to assess the early cost-effectiveness of new diagnostic tests in the early diagnosis of rheumatoid arthritis | Leander R. Buisman, Jolanda J. Luime, Mark Oppe, Johanna M. W. Hazes, Maureen P. M. H. Rutten-van Mölken | Arthritis Research & Therapy | 2016 |
| 18 | Cost Effectiveness of Human Papillomavirus-16/18 Genotyping in Cervical Cancer Screening | Warner K. Huh, Erin Williams, Joice Huang, Tommy Bramley, Nick Poulios | Applied Health Economics and Health Policy | 2015 |
| 19 | Cascade screening based on genetic testing is cost-effective: Evidence for the implementation of models of care for familial hypercholesterolemia | Zanfina Ademi, Gerald F. Watts, Jing Pang, Eric J. G. Sijbrands, Frank M. van Bockxmeer, Peter O’Leary, Elizabeth Geelhoed, Danny Liew | Journal of Clinical Lipidology | 2014 |
| 20 | Real-world cost-effectiveness of pharmacogenetic screening for epilepsy treatment | Zhibin Chen, Danny Liew, Patrick Kwan | Neurology | 2016 |
| 21 | Cost-Effectiveness Analysis of Screening for KRAS and BRAF Mutations in Metastatic Colorectal Cancer | Ajay S. Behl, Katrina A. B. Goddard, Thomas J. Flottemesch, David Veenstra, Richard T. Meenan Jennifer S. Lin, Michael V. Maciosek | Journal of the National Cancer Institute | 2012 |
| 22 | Cost-effectiveness of HLA-B*1502 genotyping in adult patients with newly diagnosed epilepsy in Singapore | Di Dong, Cynthia Sung, Eric Andrew Finkelstein | Neurology | 2012 |
| 23 | Cost–effectiveness analysis of genotyping for HLA-B*5801 and an enhanced safety program in gout patients starting allopurinol in Singapore | Di Dong, Wei-Chuen, Tan-Koi, Gim Gee Teng, Eric Finkelstein, Cynthia Sung | Pharmacogenomics | 2015 |
| 24 | Upfront Genotyping of DPYD*2A to Individualize Fluoropyrimidine Therapy: A Safety and Cost Analysis | Maarten J. Deenen, Didier Meulendijks, Annemieke Cats, Marjolein K. Sechterberger, Johan L. Severens, Henk Boot, Paul H. Smits, Hilde Rosing, Caroline M.P.W. Mandigers, Marcel Soesan, Jos H. Beijnen, Jan H.M. Schellens | Journal of Clinical Oncology | 2015 |
| 25 | Economic evaluation of genetic screening for Lynch syndrome in Germany | Franziska Severin, Björn Stollenwerk, Elke Holinski-Feder, Elisabeth Meyer, Volker Heinemann, Clemens Giessen-Jung, Wolf Rogowski | Genetics in Medicine | 2015 |
| 26 | Economic Evaluation of Using a Genetic Test to Direct Breast Cancer Chemoprevention in White Women with a Previous Breast Biopsy | Linda E. Green, Tuan A. Dinh, David A. Hinds, Bryan L. Walser, Richard Allman | Applied Health Economics and Health Policy | 2014 |
| 27 | Thiopurine S-Methyltranferase Testing in Idiopathic Pulmonary Fibrosis: A Pharmacogenetic Cost-Effectiveness Analysis | Jared T. Hagaman, Brent W. Kinder, Mark H. Eckman | Lung | 2010 |
| 28 | A cost-effectiveness model of genetic testing for the evaluation of families with hypertrophic cardiomyopathy | Jodie Ingles,Julie McGaughran, Paul A Scuffham, John Atherton, Christopher Semsarian | Heart | **2012** |
| 29 | BRAF mutation in papillary thyroid cancer: A cost-utility analysis of preoperative testing | Wayne S. Lee, Barnard J. A. Palmer, Arturo Garcia, Vincent E. Chong, Terrence H. Liu | Surgery | 2014 |
| 30 | Cost-effectiveness analysis of EGFR mutation testing and gefitinib as first-line therapy for non-small cell lung cancer | Yusuke Naritaa, Yukiko Matsushimaa, Takeru Shiroiwab, Koji Chibac, Yoichi Nakanishid, Tatsuo Kurokawaa, Hisashi Urushihara | Lung Cancer | 2015 |
| 31 | Cost-Effectiveness of Multiplexed Predictive Biomarker Screening in Non-Small Cell Lung Cancer | Dorothy Romanus, Stephanie Cardarella, David Cutler, Mary Beth Landrum, Neal I. Lindeman, G. Scott Gazelle | Journal of Thoracic Oncology | 2015 |
| 32 | Cost-effectiveness Analysis of UGT1A1 Genetic Testing to Inform Antiretroviral Prescribing in HIV Disease | Bruce R. Schackman, David W. Haas, Jessica E. Becker, Bethany K., Berkowitz, B.A., Paul E. Sax, Eric S. Daar, Heather J. Ribaudo, Kenneth A. Freedberg | Antiviral Therapy | 2013 |
| 33 | The Cost Effectiveness of Genetic Testing for CYP2C19 Variants to Guide Thienopyridine Treatment in Patients with Acute Coronary Syndromes | Laura Panattoni, Paul M. Brown, Braden Te Ao, Mark Webster, Patrick Gladding | Pharmacoeconomics | 2012 |
| 34 | Cost Effectiveness of Targeted High-dose Atorvastatin Therapy Following Genotype Testing in Patients with Acute Coronary Syndrome | Anju Parthan, Kevin J. Leahy, Amy K. O’Sullivan, Olga A. Iakoubova, Lance A. Bare, James J. Devlin, Milton C. Weinstein | Pharmacoeconomics | 2013 |
| 35 | Cost-utility analysis of genotype-guided antiplatelet therapy in patients with moderate-to high risk acute coronary syndrome and planned percutaneous coronary intervention | Vardhaman Patel, Fang-Ju Lin, Olaitan Ojo, Sapna Rao, Shengsheng YU, Lin Zhan, Daniel R. Touchette | Pharmacy Practice | 2014 |
| 36 | Cost-Effectiveness of Testing for Breast Cancer Susceptibility Genes | Margaret L. Holland, Alissa Huston, Katia Noyes | Value in Health | 2009 |
| 37 | Genetic Screening for the Predisposition to Venous Thromboembolism: A Cost-Utility Analysis of Clinical Practice in the Italian HealthCare System | Amelia Compagni, Alessia Melegaro, Rosanna Tarricone | Value in Health | 2013 |
| 38 | Cost-effectiveness of screening for HLA-A*31:01 prior to initiation of carbamazepine in epilepsy | Catrin O. Plumpton, Vincent L. M. Yip, Ana Alfirevic, Anthony G. Marson, Munir Pirmohamed, Dyfrig A. Hughes | Epilepsia | 2015 |
| 39 | Cost-Effectiveness Analysis of HLA-B*5801 Testing in Preventing Allopurinol-Induced SJS/TEN in Thai Population | Surasak Saokaew, Wichittra Tassaneeyakul, Ratree Maenthaisong, Nathorn Chaiyakunapruk | Plos One | 2014 |
| 40 | Cost-Effectiveness Analysis of Different Genetic Testing Strategies for Lynch Syndrome in Taiwan | Ying-Erh Chen, Sung-Shuo Kao, Ren-Hua Chung | Plos One | 2016 |
| 41 | Economic Utility of a Blood-Based Genomic Test for the Assessment of Patients with Symptoms Suggestive of Obstructive Coronary Artery Disease | Louis I. Hochheiser, Jessie L. Juusola, Mark Monane, Joseph A. Ladapo | Population Health Management | 2014 |
| 42 | Cost-Effectiveness Model of Use of Genetic Testing as an Aid in Assessing the Likely Benefit of Aspirin Therapy for Primary Prevention of Cardiovascular Disease | Dov Shiffman, Katherine Slawsky, Lauren Fusfeld, James J. Devlin, Thomas F. Goss | Clinical Therapeutics | 2012 |
| 43 | Detecting Germline PTEN Mutations Among At-Risk Patients With Cancer: An Age- and Sex-Specific Cost-Effectiveness Analysis | Joanne Ngeow, Chang Liu, Ke Zhou, Kevin D. Frick, David B. Matchar, Charis Eng | Journal of Clinical Oncology | 2015 |
| 44 | Cost-effectiveness of KRAS testing in metastatic colorectal cancer patients in the United States and Germany | Arthi Vijayaraghavan, Molly B. Efrusy, Burkhard Goke, Thomas Kirchner, Christopher C. Santas, Richard M. Goldberg | International Journal of Cancer | 2011 |
| 45 | Cost minimization of HLA-B*1502 screening before prescribing carbamazepine in Thailand | Somsak Tiamkao, Jukrapope Jitpimolmard, Kittisak Sawanyawisuth, Suthipun Jitpimolmard | International Journal of Clinical Pharmacy | 2013 |
| 46 | A Cost-Effectiveness Evaluation of Germline BRCA1 and BRCA2 Testing in UK Women with Ovarian Cancer | Anthony Eccleston,Mmath Stat, Anthony Bentley, Matthew Dyer, Ann Strydom, Wim Vereecken, Angela George, Nazneen Rahman | Value in Health | 2017 |
| 47 | CYP2C19 LOF and GOF-Guided Antiplatelet Therapy in Patients with Acute Coronary Syndrome: A Cost-Effectiveness Analysis | Minghuan Jiang, Joyce H. S. You | Cardiovascular Drugs and Therapy | 2016 |
| 48 | Cost-effectiveness Analysis for Genotyping before Allopurinol Treatment to Prevent Severe Cutaneous Adverse Drug Reactions | Ching-Hua Ke, Wen-Hung Chung, Yen-Hsia Wen, Yaw-Bin Huang, Hung-Yi Chuang, You-Lin Tain, Yu-Ching Lily Wang, Cheng-Chih Wu and Chien-Ning Hsu | The Journal of Rheumatology | 2017 |
| 49 | A Multigene Test Could Cost-Effectively Help Extend Life Expectancy for Women at Risk of Hereditary Breast Cancer | Yonghong Li, Andre R. Arellano,Lance A. Bare, Richard A. Bender, Charles M. Strom, James J. Devlin | Value in Health | 2017 |
| 50 | The cost-effectiveness of genetic testing strategies for Lynch syndrome among newly diagnosed patients with colorectal cancer | Mercy Mvundura, Scott D. Grosse, Heather Hampel, Glenn E. Palomaki | Genetics in Medicine | 2010 |
| 51 | Economic issues involved in integrating genomic testing into clinical care: the case of genomic testing to guide decision-making about chemotherapy for breast cancer patients | Patricia Marino, Carole Siani, Francois Bertucci, Henri Roche, Anne-Laure Martin, Patrice Viens, Valerie Seror | Breast Cancer Research and Treatment | 2011 |
| 52 | Clinical and Economic Aspects of KRAS Mutational Status as Predictor for Epidermal Growth Factor Receptor Inhibitor Therapy in Metastatic Colorectal Cancer Patients | Robert Königsberg, Wolfgang Hulla, Martin Klimpfinger, Angelika Reiner-Concin, Tanja Steininger, Wilfried Büchler, Robert Terkola, Christian Dittrich | Oncology | **2011** |
| 53 | Cost Effectiveness of Gene Expression Profiling for Early Stage Breast Cancer | Mo Yang, Suja Rajan, Amalia M. Issa | Cancer | 2012 |
| 54 | Cost effectiveness of pharmacogenetic testing for UGT1A1 before irinotecan administration for metastatic colorectal cancer | Heather T Gold, Michael J Hall, Victoria Blinder, Bruce R Schackman | Cancer | 2009 |
| 55 | Cost-effectiveness of UGT1A1 genotyping in second-line, high-dose, once every 3 weeks irinotecan monotherapy treatment of colorectal cancer | Marko Obradovic, Ales Mrhar, Mitja Kos | Pharmacogenomics | 2008 |
| 56 | Cost-Effectiveness of Pharmacogenetics-Guided Warfarin Therapy vs. Alternative Anticoagulation in Atrial Fibrillation | J Pink, M Pirmohamed, S Lane, DA Hughes | Clinical Pharmacology & Therapeutics | 2014 |
| 57 | Genetic Testing in Combination with Preventive Donepezil Treatment for Patients with Amnestic Mild Cognitive Impairment | Sandjar Djalalov, Jean Yong, Jaclyn Beca, Sandra Black, Gustavo Saposnik, Zahra Musa, Katherine Siminovitch, Myla Moretti, Jeffrey S. Hoch | Molecular Diagnosis & Therapy | **2012** |
| 58 | Cost-effectiveness analysis of HLA-B*5701 typing in the prevention of hypersensitivity to abacavir in HIV+patients in Spain | Diana Nieves Calatrava, O´ scar dela Calle-Martı´n, Jose´ A. Iribarren-Loyarte, Antonio Rivero-Roma´n, Laura Garcı´a-Bujalance, Isabel Pe´ rez-Escolano, Max Brosa-Riestra | Enfermedades Infecciosas y Microbiología Clínica | 2010 |
| 59 | Economic Evaluation of Genomic Test–Directed Chemotherapy for Early-Stage Lymph Node–Positive Breast Cancer | Peter S. Hall, Christopher McCabe, Robert C. Stein, David Cameron | Journal of the National Cancer Institute | **2012** |
| 60 | The cost-effectiveness of HLA-BM5701 genetic screening to guide initial antiretroviral therapy for HIV | Bruce R. Schackman, Callie A. Scottb, Rochelle P. Walensky, Elena Losina, Kenneth A. Freedberg, Paul E. Sax | AIDS | 2008 |
| 61 | Economic Analysis of Thrombo inCode, a Clinical–Genetic Function for Assessing the Risk of Venous Thromboembolism | C. Rubio-Terre´s, J. M. Soria, P. E. Morange, J. C. Souto, P. Suchon, J. Mateo, N. Saut, D. Rubio-Rodrı´guez, J. Sala, A. Gracia, S. Pich, E. Salas | Applied Health Economics and Health Policy | 2015 |
| 62 | KRAS and BRAF Mutation Analysis in Metastatic Colorectal Cancer: A Cost-effectiveness Analysis from a Swiss Perspective | Patricia R. Blank, Holger Moch, Thomas D. Szucs, Matthias Schwenkglenks | Clinical Cancer Research | 2011 |
| 63 | The Potential Clinical and Economic Outcomes of Pharmacogenomic Approaches to EGFR-Tyrosine Kinase Inhibitor Therapy in Non–Small-Cell Lung Cancer | Josh J. Carlson, Louis P. Garrison, Scott D. Ramsey, David L. Veenstra | Value in Health | 2009 |
| 64 | Economic Efficiency of Genetic Screening to Inform the Use of Abacavir Sulfate in the Treatment of HIV | Teresa L. Kauf, Raymond A. Farkouh, Stephanie R. Earnshaw, Maria E. Watson, Penny Maroudas, Mike G. Chambers | Pharmacoeconomics | 2010 |
| 65 | An economic assessment of genetic testing for familial adenomatous polyposis | Olry de Labry Lima, L. Sordo del Castillo, L. García Mochón, D. Epstein, C. Bermúdez Tamayo, R. Villegas Portero | Revista Espanola De Enfermedades Digestivas | 2008 |
| 66 | The cost-effectiveness of prenatal screening for spinal muscular atrophy | Sarah E. Little, Vanitha Janakiraman, Anjali Kaimal, Thomas Musci, Jeffrey Ecker, Aaron B. Caughey | American Journal of Obstetrics & Gynecology | 2010 |
| 67 | Pharmacoeconomic Analyses of Azathioprine, Methotrexate and Prospective Pharmacogenetic Testing for the Management of Inflammatory Bowel Disease | Virginia L. Priest, Evan J. Begg, Sharon J. Gardiner, Christopher M.A. Frampton, Richard B. Gearry, Murray L. Barclay, David W.J. Clark, Paul Hansen | Pharmacoeconomics | 2006 |
| 68 | Economic evaluation of HLA-B*15:02 screening for carbamazepine-induced severe adverse drug reactions in Thailand | Waranya Rattanavipapong, Tanunya Koopitakkajorn, Naiyana Praditsitthikorn, Surakameth Mahasirimongkol, Yot Teerawattananon | Epilepsia | 2013 |
| 69 | The Cost-Effectiveness of a Pharmacogenetic Test: A Trial-Based Evaluation of TPMT Genotyping for Azathioprine | Alexander J. Thompson, William G. Newman, Rachel A. Elliott, Stephen A. Roberts, Karen Tricker, Katherine Payne | Value in Health | 2014 |
| 70 | Predictive genetic testing of first degree relatives of mutation carriers is a cost-effective strategy in preventing hereditary nonpolyposis colorectal cancer in Singapore | Vivian Wei Wang, Poh Koon Koh, Wai Leng Chow, Jeremy Fung Yen Lim | Familial Cancer | 2012 |
| 71 | Next-Generation Sequencing Panels for the Diagnosis of Colorectal Cancer and Polyposis Syndromes: A Cost-Effectiveness Analysis | Carlos J. Gallego, Brian H. Shirts, Caroline S. Bennette, Greg Guzauskas, Laura M. Amendola, Martha Horike-Pyne, Fuki M. Hisama, Colin C. Pritchard, William M. Grady, Wylie Burke, Gail P. Jarvik, and David L. Veenstra | Journal of Clinical Oncology | 2015 |
| 72 | A Policy Model to Evaluate the Benefits, Risks and Costs of Warfarin Pharmacogenomic Testing | Lisa M. Meckley, James M. Gudgeon, Jeffrey L. Anderson, Marc S. Williams, David L. Veenstra | Pharmacoeconomics | 2010 |
| 73 | A cost analysis of a cancer genetic service model in the UK | Ingrid Slade, Helen Hanson, Angela George, Kelly Kohut, Ann Strydom, Sarah Wordsworth, Nazneen Rahman | Journal of Community Genetics | 2016 |
| 74 | Cost-effectiveness of pharmacogenetic-guided dosing of warfarin in the United Kingdom and Sweden | TI Verhoef, WK Redekop, S Langenskiold, F Kamali, M Wadelius, G Burnside, A-H Maitland-van der Zee, DA Hughes, M Pirmohamed | The Pharmacogenomics Journal | 2016 |
| 75 | Cost–effectiveness of pharmacogenetic-guided dosing of phenprocoumon in atrial fibrillation | Talitha I Verhoef, William K Redekop, David L Veenstra, Rahber Thariani, Peter A Beltman, Rianne MF van Schie, Anthonius de Boer, Anke-Hilse Maitlandvan der Zee | Pharmacogenomics | 2013 |
| 76 | Economic evaluation of a pharmacogenetic dosing algorithm for coumarin anticoagulants in The Netherlands | Talitha I Verhoef, William K Redekop, Anthonius de Boer, Anke Hilse Maitlandvan der Zee | Pharmacogenomics | 2015 |
| 77 | Economic evaluation of pharmacogenomic-guided warfarin treatment for elderly Croatian atrial fibrillation patients with ischemic stroke | Christina Mitropoulou, Vasilios Fragoulakis, Nada Bozina, Athanassios Vozikis, Svjetlana Supe, Tamara Bozina, Zdravka Poljakovic, Ron H van Schaik, George P Patrinos | Pharmacogenomics | **2015** |
| 78 | A Value‑Based Medicine cost‑utility analysis of genetic testing for neovascular macular degeneration | Gary C. Brown, Melissa M. Brown, Heidi B. Lieske, Philip A. Lieske, Kathryn S. Brown | International Journal of Retina and Vitreous | 2015 |
| 79 | Economic analysis of pharmacogenomic-guided clopidogrel treatment in Serbian patients with myocardial infarction undergoing primary percutaneous coronary intervention | Christina Mitropoulou, Vasilios Fragoulakis, Ljiljana B Rakicevic, Mirjana M Novkovic, Athanassios Vozikis, Dragan M Matic, Nebojsa M Antonijevic, Dragica P Radojkovic, Ron H van Schaik, George P Patrinos | Pharmacogenomics | 2016 |
| 80 | Cost-effectiveness analysis of MTHFR polymorphism screening by polymerase chain reaction in Korean patients with rheumatoid arthritis receiving methotrexate | Seong-Kyu Kim, Jae-Bum Jun, Ahmed El-Sohemy, Sang-Cheol Bae | The Journal of Rheumatology | **2006** |
| 81 | Cost-effectiveness of pharmacogenetic testing to predict treatment response to angiotensin-converting enzyme inhibitor | Madlaina Costa-Scharplatz, Antoinette D.I. van Asselt, Lucas M. Bachmann, Alfons G.H. Kessels, Johan L. Severens | Pharmacogenetics and Genomics | 2007 |
| 82 | Cost-utility analysis of genetic screening in families of patients with germline MUTYH mutations | Maartje Nielsen, Frederik J Hes, Hans FA Vasen, Wilbert B van den Hout | BMC Medical Genetics | 2007 |
| 83 | Cost-effectiveness of Population Screening for BRCA Mutations in Ashkenazi Jewish Women Compared With Family History–Based Testing | Ranjit Manchanda, Rosa Legood, Matthew Burnell, Alistair McGuire, Maria Raikou, Kelly Loggenberg, Jane Wardle, Saskia Sanderson, Sue Gessler, Lucy Side, Nyala Balogun, Rakshit Desai, Ajith Kumar, Huw Dorkins, Yvonne Wallis, Cyril Chapman, Rohan Taylor, Chris Jacobs, Ian Tomlinson, Uziel Beller, Usha Menon, Ian Jacobs | Journal of the National Cancer Institute | 2015 |
| 84 | Clinical relevance and cost-effectiveness of HLA genotyping in children with Type 1 diabetes mellitus in screening for coeliac disease in the Netherlands | J. Elias, J. J. G. Hoorweg-Nijman, W. A. Balemans | Diabetic Medicine | 2014 |
| 85 | Financial Analysis of CYP2C19 Genotyping in Patients Receiving Dual Antiplatelet Therapy Following Acute Coronary Syndrome and Percutaneous Coronary Intervention | Samuel G. Johnson, Don Gruntowicz, Theresa Chua, Robert J. Morlock | Journal of Managed Care & Specialty Pharmacy | 2015 |
| 86 | Cost Effectiveness of Personalized Therapy for First-Line Treatment of Stage IV and Recurrent Incurable Adenocarcinoma of the Lung | By Elizabeth A. Handorf, Sean McElligott, Anil Vachani, Corey J. Langer, Mirar Bristol Demeter, Katrina Armstrong, David A. Asch | Journal of Oncology Practice | 2012 |
| 87 | A Cost Savings Approach to SPRED1 Mutational Analysis in Individuals at Risk for Neurofibromatosis Type 1 | Talia M. Muram, David A. Stevenson, Sarah Watts-Justice, David H. Viskochil, John C. Carey, Rong Mao, Brian Jackson | American Journal of Medical Genetics | **2013** |
| 88 | Does Pharmacogenetic Testing for CYP450 2D6 and 2C19 Among Patients with Diagnoses within the Schizophrenic Spectrum Reduce Treatment Costs? | Louise Herbild, Stig E. Andersen, Thomas Werge, Henrik B. Rasmussen and Gesche Jurgens | Basic & Clinical Pharmacology & Toxicology | 2013 |
| 89 | Cost-Effectiveness of MODY Genetic Testing: Translating Genomic Advances Into Practical Health Applications | Rochelle N. Naylor, Priya M. John, Aaron N. Winn, David Carmody, Siri AtmaW. Greeley, Louis H. Philipson, Graeme I. Bell, Elbert S. Huang | Diabetes Care | 2014 |
| 90 | Cost-Effectiveness of Genotype-Guided Warfarin Dosing for Patients With Atrial Fibrillation | Amanda R. Patrick, Jerry Avorn, Niteesh K. Choudhry | Circulation. Cardiovascular Quality and Outcomes | 2009 |
| 91 | Health Benefits and Cost-Effectiveness of Primary Genetic Screening for Lynch Syndrome in the General Population | Tuan A. Dinh, Benjamin I. Rosner, James C. Atwood, C. Richard Boland, Sapna Syngal, Hans F. A. Vasen, Stephen B. Gruber, Randall W. Burt | Cancer Prevention Research | 2011 |
| 92 | Non-invasive prenatal diagnosis for cystic fibrosis: detection of paternal mutations, exploration of patient preferences and cost analysis | Melissa Hill, Philip Twiss, Talitha I. Verhoef, Suzanne Drury, Fiona McKay, Sarah Mason, Lucy Jenkins, Stephen Morris, Lyn S. Chitty | Prenatal Diagnosis | 2015 |
| 93 | Human epidermal growth factor receptor 2 expression in early breast cancer patients: a Swiss cost–effectiveness analysis of different predictive assay strategies | Patricia R. Blank, Matthias Schwenkglenks, Holger Moch, Thomas D. Szucs | Breast Cancer Research and Treatment | 2010 |
| 94 | Strategies to Identify the Lynch Syndrome Among Patients With Colorectal Cancer: A Cost-Effectiveness Analysis | Uri Ladabaum, Grace Wang, Jonathan Terdiman, Amie Blanco, Miriam Kuppermann, C. Richard Boland, James Ford, Elena Elkin, Kathryn A. Phillips | Annals of Internal Medicine | 2011 |
| 95 | Cost-effectiveness analysis of carrier and prenatal genetic testing for X-linked hemophilia | Meng-Che Tsai, Chao-Neng Cheng, Ru-Jay Wang , Kow-Tong Chen, Mei-Chin Kuo, Shio-Jean Lin | Journal of the Formosan Medical Association | 2015 |
| 96 | Cost-effectiveness of carrier screening for cystic fibrosis in Australia | Richard Norman, Kees van Gool, Jane Hall, Martin Delatycki, John Massie | Journal of Cystic Fibrosis | 2012 |

| Supplementary Table 2. Characteristics of economic evaluation analyses | | | | | |
| --- | --- | --- | --- | --- | --- |
| r/n | **Economic evaluation analysis** | **Cohort analysis** | **Outcome measurement** | **Cost measurement** | **Economic evaluation model** |
| 1 | Cost-utility | Hypothetical | QALYs | Direct medical costs,  Direct non-medical costs,  Indirect costs | Decision tree,  Markov model |
| 2 | Cost-utility | Hypothetical | QALYs | Direct medical costs | Decision tree |
| 3 | Cost-effectiveness | Hypothetical | Risk of death from  myelosuppression | Direct medical costs | Decision tree |
| 4 | Cost-utility,  Cost-effectiveness | Hypothetical | LYs, QALYs | Direct medical costs | Markov model |
| 5 | Cost-utility,  Cost-effectiveness | Hypothetical | Riskofdeathfrom  cervical cancer,  QALYs | Direct medical costs,  Indirect costs | Markov model |
| 6 | Cost-effectiveness | Hypothetical | LYs | Direct medical costs | Flow chart |
| 7 | Cost-utility | Hypothetical | QALYs | Direct medical costs | Decision tree,  Markov model |
| 8 | Cost-utility,  Cost-effectiveness | Hypothetical | LYs, QALYs | Direct medical costs | Decision tree,  Markov model |
| 9 | Cost-utility,  Cost-effectiveness | Hypothetical | LYs, QALYs | Direct medical costs | Decision tree,  Markov model |
| 10 | Cost-effectiveness | Hypothetical | LYs | Direct medical costs | - |
| 11 | Cost-utility,  Cost-effectiveness | Hypothetical | Probabilityof end-stage complications of type 1 diabetes, QALYs | Direct medical costs,  Indirect costs | Decision tree,  Markov model |
| 12 | Cost-effectiveness | Hypothetical | LYs | Direct medical costs | Decision tree,  Markov model |
| 13 | Cost-utility,  Cost-effectiveness | Hypothetical | Cancer deaths, QALYs | Direct medical costs | Discrete event  simulation model |
| 14 | Cost-effectiveness | Hypothetical | Number offebrileneutropenia avoided | Direct medical costs | Decision tree |
| 15 | Cost-utility | Hypothetical | Cost saving, QALYs | Direct medical costs | Markov model |
| 16 | Cost-utility,  Cost-effectiveness | Hypothetical | Adjuvant chemotherapy, Recurrence,  QALYs | Direct medical costs,  Direct non-medical costs,  Indirect costs | Decision tree,  Markov model |
| 17 | Cost-utility,  Cost-effectiveness | Retrospective | Test results, QALYs | Direct medical costs,  Indirect costs | Decision tree,  Markov model |
| 18 | Cost-utility,  Cost-effectiveness | Hypothetical | Cervical cancer incidence, Mortality,  QALYs, LYs | Direct medical costs | Decision tree,  Markov model |
| 19 | Cost-utility,  Cost-effectiveness | Hypothetical | LYs, QALYs | Direct medical costs | Decision tree,  Markov model |
| 20 | Cost-utility | Retrospective | QALYs | Direct medical costs | Decision tree |
| 21 | Cost-effectiveness | Hypothetical | LYs | Direct medical costs | Decision tree,  Markov model |
| 22 | Cost-utility | Hypothetical | QALYs | Direct medical costs | Decision tree |
| 23 | Cost-utility | Hypothetical | QALYs | Direct medical costs | Decision tree |
| 24 | Cost-effectiveness,  Cost-minimization | Prospective | Toxicity, Drug-induced death,  Total treatment costs | Direct medical costs | Decision tree |
| 25 | Cost-effectiveness | Hypothetical | LYs | Direct medical costs | Decision tree,  Markov model |
| 26 | Cost-utility,  Cost-effectiveness | Hypothetical | Breast cancer incidence rate,  Death rate, QALYs | Direct medical costs | Discrete event  simulation model |
| 27 | Cost-utility | Hypothetical | QALYs | Direct medical costs | Decision tree |
| 28 | Cost-utility,  Cost-effectiveness | Hypothetical | LYs, QALYs | Direct medical costs | Decision tree,  Markov model |
| 29 | Cost-utility | Hypothetical | QALYs | Direct medical costs,  Indirect costs | Decision tree,  Markov model |
| 30 | Cost-utility | Hypothetical | QALYs | Direct medical costs | Decision tree,  Markov model |
| 31 | Cost-utility,  Cost-effectiveness | Hypothetical | LYs, QALYs | Direct medical costs,  Direct non-medical costs,  Indirect costs | Flow chart |
| 32 | Cost-utility | Hypothetical | QALYs | Direct medical costs | Decision tree |
| 33 | Cost-utility | Retrospective | QALYs | Direct medical costs | Decision tree |
| 34 | Cost-utility,  Cost-effectiveness | Hypothetical | Cardiovascular event rates,  LYs, QALYs | Direct medical costs | Decision tree,  Markov model |
| 35 | Cost-utility,  Cost-effectiveness | Hypothetical | LYs, QALYs | Direct medical costs | Decision tree |
| 36 | Cost-utility | Hypothetical | QALYs | Direct medical costs | Semi-Markov model |
| 37 | Cost-utility | Hypothetical | QALYs | Direct medical costs | Decision tree |
| 38 | Cost-utility,  Cost-effectiveness | Hypothetical | Seizure-freeyears,  Cutaneous ADR avoided,  LYs, QALYs | Direct medical costs | Decision tree,  Markov model |
| 39 | Cost-utility,  Cost-effectiveness | Hypothetical | SJS/TEN incidence,  Death in SJS/TEN cases, QALYs | Direct medical costs,  Direct non-medical costs | Decision tree,  Markov model |
| 40 | Cost-effectiveness | Hypothetical | LYs | Direct medical costs,  Other costs | Decision tree,  Markov model |
| 41 | Financial analysis | Hypothetical | Total treatment costs | Direct medical costs | Decision tree |
| 42 | Cost-utility,  Cost-effectiveness | Hypothetical | Cardiovascular events avoided,  Bleeding events, QALYs | Direct medical costs | Flow chart |
| 43 | Cost-utility | Hypothetical | QALYs | Direct medical costs,  Indirect costs | Decision tree |
| 44 | Cost-effectiveness | Hypothetical | Cost savings/patient,  Total cost/patients, LYs | Direct medical costs | Decision tree,  Markov model |
| 45 | Cost-minimization analysis | Retrospective | Total treatment cost | Direct medical costs | Flow chart |
| 46 | Cost-utility,  Cost-effectiveness | Hypothetical | Deaths,  Ovarian/Breast cancer cases,  QALYs | Direct medical costs | Flow chart |
| 47 | Cost-utility,  Cost-effectiveness | Hypothetical | Nonfatal myocardialinfarction,  Nonfatal stroke, Cardiovasculardeath,  Stentthrombosis,  Majorbleeding, QALYs | Direct medical costs | Decision tree,  Markov model |
| 48 | Cost-utility,  Cost-effectiveness | Retrospective | LYs, QALYs | Direct medical costs | Decision tree |
| 49 | Cost-utility,  Cost-effectiveness | Hypothetical | LYs, QALYs | Direct medical costs | Decision tree,  Markov model |
| 50 | Cost-utility,  Cost-effectiveness | Hypothetical | LYS, QALYs | Direct medical costs | Flow chart |
| 51 | Cost-minimization analysis | Retrospective | Total treatment costs | Direct medical costs | Decision tree |
| 52 | Financial analysis | Retrospective | Total treatment costs | Direct medical costs | - |
| 53 | Cost-utility | Hypothetical | QALYs | Direct medical costs | Decision tree,  Markov model |
| 54 | Cost-utility | Hypothetical | QALYs | Direct medical costs | Decision tree |
| 55 | Cost-effectiveness | Hypothetical | Severeneutropeniacasesavoided, LYs | Direct medical costs | Decision tree |
| 56 | Cost-utility,  Cost-effectiveness | Hypothetical | Stroke or systemic embolism,  Transient ischemic attack,  Intracranial hemorrhage, Major bleed,  Nonfatal myocardial infarction, QALYs, LΥs | Direct medical costs | Discrete event  simulation model |
| 57 | Cost-utility | Hypothetical | QALYs | Direct medical costs,  Indirect costs | Markov model |
| 58 | Cost-effectiveness | Hypothetical | Cases of hypersensitivity reactions avoided | Direct medical costs,  Direct non-medical costs | Decision tree |
| 59 | Cost-utility,  Cost-effectiveness | Hypothetical | LYs, QALYs | Direct medical costs | Decision tree,  Modified Markov model |
| 60 | Cost-utility,  Cost-effectiveness | Hypothetical | Confirmed and unconfirmed severe  systemic hypersensitivity reaction, QALYs | Direct medical costs | CEPAC model |
| 61 | Cost-utility,  Cost-effectiveness | Hypothetical | LYs, QALYs | Direct medical costs | Decision tree |
| 62 | Cost-utility | Hypothetical | QALYs | Direct medical costs | Decision tree,  Markov model |
| 63 | Cost-utility,  Cost-effectiveness | Hypothetical | LYs, QALYs | Direct medical costs | Decision tree |
| 64 | Cost-utility,  Cost-effectiveness | Hypothetical | Totaltreatmentcost/patient,  Clinically diagnosed hypersensitivity reaction/patient,  Mean life expectancy (years), QALYs | Direct medical costs,  Direct non-medical costs | Decision tree,  Discrete event  simulation model |
| 65 | Cost-utility | Hypothetical | QALYs | Direct medical costs | Markov model |
| 66 | Cost-utility,  Cost-effectiveness | Hypothetical | Cases of spinal muscular atrophy cases,  Procedure-related miscarriages,  Pregnancy terminations,QALYs | Direct medical costs,  Indirect costs | Decision tree |
| 67 | Cost-utility,  Cost-effectiveness | Hypothetical | Neutropenia events, QALYs | Direct medical costs | Decision tree |
| 68 | Cost-utility,  Cost-effectiveness | Hypothetical | LYs, QALYs, Lifetime costs | Direct medical costs,  Direct non-medical costs,  Indirect costs | Decision tree,  Markov model |
| 69 | Cost-utility | Prospective | QALYs | Direct medical costs | Flow chart |
| 70 | Cost-effectiveness | Hypothetical | LYs | Direct medical costs | Markov model |
| 71 | Cost-utility,  Cost-effectiveness | Hypothetical | LYs, QALYs | Direct medical costs | Decision tree |
| 72 | Cost-utility,  Cost-effectiveness | Hypothetical | Bleeding,  Thromboembolic events,  Deaths, QALYs | Direct medical costs | Decision tree,  Markov model |
| 73 | Financial analysis | Retrospective | Total treatment costs | Direct medical costs,  Other costs | Flow chart |
| 74 | Cost-utility,  Cost-effectiveness | Hypothetical | Bleeding,  Thromboembolic events, QALYs | Direct medical costs | Markov model |
| 75 | Cost-utility,  Cost-effectiveness | Hypothetical | Bleeding,  Thromboembolic events,  Deaths, QALYs | Direct medical costs | Decision tree,  Markov model |
| 76 | Cost-utility | Hypothetical | QALYs | Direct medical costs | Decision tree,  Markov model |
| 77 | Cost-utility | Retrospective | QALYs | Direct medical costs | Decision tree |
| 78 | Cost-utility | Hypothetical | QALYs | Direct medical costs,  Direct non-medical costs,  Indirect costs | Markov model |
| 79 | Financial analysis | Retrospective | Costs | Direct medical costs | Decision tree |
| 80 | Cost-effectiveness | Retrospective | Treatment effectiveness, Costs | Direct medical costs | Decision tree |
| 81 | Cost-effectiveness | Hypothetical | LYs, Patients without end-stage renal disease | Direct medical costs | Decision tree,  Markov model |
| 82 | Cost-utility,  Cost-effectiveness | Hypothetical | LYs, QALYs | Direct medical costs,  Direct non-medical costs,  Indirect costs | Decision tree,  Markov model |
| 83 | Cost-utility,  Cost-effectiveness | Hypothetical | LYs, QALYs | Direct medical costs | Decision tree |
| 84 | Financial analysis | Retrospective | Total treatment costs | Direct medical costs | - |
| 85 | Financial analysis | Hypothetical | Total treatment costs | Direct medical costs | - |
| 86 | Cost-utility | Hypothetical | QALYs | Direct medical costs,  Indirect costs | Decision tree |
| 87 | Financial analysis | Hypothetical | Total treatment costs | Direct medical costs | Markov model |
| 88 | Financial analysis | Retrospective | Total treatment costs | Direct medical costs | - |
| 89 | Cost-utility | Hypothetical | QALYs | Direct medical costs | Decision tree |
| 90 | Cost-utility,  Cost-effectiveness | Hypothetical | LYs, QALYs | Direct medical costs | Decision tree,  Markov model |
| 91 | Cost-utility,  Cost-effectiveness | Hypothetical | QALYs, Reduction of colorectal cancer incidence, Reduction of endometrial cancer incidence, LYs | Direct medical costs | Discrete event  simulation model |
| 92 | Financial analysis | Prospective | Total costsof non-invasive prenatal diagnosis,Total costsof invasive prenatal diagnosis | Direct medical costs | Decision tree |
| 93 | Cost-utility | Hypothetical | QALYs | Direct medical costs | Markov model |
| 94 | Cost-effectiveness | Hypothetical | LYs | Direct medical costs | Markov model |
| 95 | Cost-effectiveness | Hypothetical | LYs | Direct medical costs,Direct non-medical costs,Indirect costs | Decision tree |
| 96 | Cost-effectiveness | Hypothetical | Cystic fibrosis infants/ 100,000 births | Direct medical costs | Decision tree |

| Supplementary Table 3. Characteristics of individualized interventions | | | | |
| --- | --- | --- | --- | --- |
| r/n | **Disease** | **ICD** | **Gene/Genomic test** | **Country** |
| 1 | Familial hypercholesterolemia | Ε78.9 | *LDLR, APOB* | USA |
| 2 | Advanced adenocarcinoma  of the lung | C34.9 | *EGFR* | Singapore |
| 3 | Acute lymphoblastic leukemia | C91.0 | *TPMT* | Canada |
| 4 | Colorectal cancer | C18.9 | *KRAS* | Japan |
| 5 | Cervical cancer | C53.9 | HPV DNA | South Africa |
| 6 | Cystic fibrosis | Ε84.9 | *CFTR* | The Netherlands |
| 7 | Acute coronary syndrome | Ι25.9 | *CYP2C19* | USA |
| 8 | Breast cancer | C50.9 | MammaPrint^®^, Oncotype DX^®^ | Spain |
| 9 | Long-QT syndrome | I45.8 | *KCNQ1, HERG, SCN5A, ANK2, KCNE1* | USA |
| 10 | Familial hypercholesterolemia | Ε78.9 | *LDLR* | Spain |
| 11 | Neonatal diabetes | P70.2 | *KCNJ11, ABCC8* | USA |
| 12 | Hypertrophic cardiomyopathy | I42.1 | *MYH7, MYBPC3, TNNT2, TNNI3* | United Kingdom |
| 13 | Breast cancer | C50.9 | *FGFR2, TOX3, MAP3K1,*  rs13387042, rs13281615,  *FGF10, LSP1-H19* | USA |
| 14 | Drug-induced neutropenia | C18.9 | *UGT1A1*28* | France |
| 15 | Atrial fibrillation | I48 | 4q25chromosome | USA |
| 16 | Breast cancer | C50.9 | Oncotype DX^®^ | Japan |
| 17 | Rheumatoid arthritis | Μ05 | - | The Netherlands |
| 18 | Cervical cancer | C53.9 | HPV-16/18 DNA | USA |
| 19 | Familial hypercholesterolemia | E78.9 | *LDLR, APOB* | Australia |
| 20 | Epilepsy | G40.9 | *HLA-B*15:02* | China |
| 21 | Colorectal cancer | C18.9 | *KRAS, BRAF* | USA |
| 22 | Epilepsy | G40.9 | *HLA-B*15:02* | Singapore |
| 23 | Gout | Μ10 | *HLA-B*58:01* | Singapore |
| 24 | Colorectal cancer,  Breast cancer,  Gastroesophageal cancer | C18.9, C50.9, C15.9 | *DPYD*2A* | The Netherlands |
| 25 | Colorectal cancer (Lynch syndrome) | C18.9 | *MLH1, MSH2, MSH6, PMS2, BRAF* | Germany |
| 26 | Breast cancer | C50.9 | BREVAGenTM^®^ | USA |
| 27 | Idiopathic pulmonary fibrosis | J84.1 | *TPMT* | USA |
| 28 | Hypertrophic cardiomyopathy | I42.1 | HCM genes | Australia |
| 29 | Papillary thyroid cancer | C73 | *BRAF* | USA |
| 30 | Non-small cell lung cancer | C34.9 | *EGFR* | Japan |
| 31 | Non-small cell lung cancer | C34.9 | *EGFR, ALK* | USA |
| 32 | Acquired immune deficiency syndrome (AIDS) | B24 | *UGT1A1*28* | USA |
| 33 | Acute coronary syndrome | Ι25.9 | *CYP2C19* | New Zealand |
| 34 | Acute coronary syndrome | Ι25.9 | *KIF6* | USA |
| 35 | Acute coronary syndrome | Ι25.9 | *CYP2C19* | USA |
| 36 | Breast cancer, Ovarian cancer | C50.9 | *BRCA1/2* | USA |
| 37 | Venous thromboembolism | I82.9 | Factor V Leiden, factor II (prothrombin) | Italy |
| 38 | Epilepsy | G40.9 | *HLA-A*31:01* | United Kingdom |
| 39 | Gout | Μ10 | *HLA-B*58:01* | Thailand |
| 40 | Colorectal cancer (Lynch syndrome) | C18.9 | *MLH1, MSH2, MSH6, PMS2, BRAF* | China |
| 41 | Obstructive coronary artery disease | Ι25.9 | Corus CAD^®^ | USA |
| 42 | Myocardial infarction, Stroke, Gastrointestinal bleeding events | I21.9, I63.9, R58, K92.2 | *LPA* | USA |
| 43 | Cowden syndrome | Q85.8 | *PTEN* | USA |
| 44 | Colorectal cancer | C18.9 | *KRAS* | USA, Germany |
| 45 | Epilepsy | G40.9 | *HLA-B*15:02* | Thailand |
| 46 | Ovarian cancer | C56 | *BRCA1/2* | United Kingdom |
| 47 | Acute coronary syndrome | I25.9 | *CYP2C19* | China |
| 48 | Gout | M10 | *HLA-B*58:01* | China (Taiwan) |
| 49 | Breast cancer, Ovarian cancer | C50.9 | *BRCA1/2, PALB2, TP53, CDH1, STK11, PTEN* | USA |
| 50 | Colorectal cancer (Lynch syndrome) | C18.9 | *MLH1, MSH2, MSH6, PMS2, BRAF* | USA |
| 51 | Breast cancer | C50.9 | 189-gene signature | France |
| 52 | Colorectal cancer | C18.9 | *KRAS* | Austria |
| 53 | Breast cancer | C50.9 | MammaPrint^®^, Oncotype DX^®^ | USA |
| 54 | Colorectal cancer | C18.9 | *UGT1A1*28* | USA |
| 55 | Colorectal cancer | C18.9 | *UGT1A1*28* | USA |
| 56 | Atrial fibrillation | I48 | *VKORC1, CYP2C9* | United Kingdom |
| 57 | Amnestic mild cognitive impairment | G31.84 | *APOE* | Canada |
| 58 | Acquired immune deficiency syndrome (AIDS) | B24 | *HLA-B*57:01* | Spain |
| 59 | Breast cancer | C50.9 | Oncotype DX^®^ | United Kingdom |
| 60 | Acquired immune deficiency syndrome (AIDS) | B24 | *HLA-B*57:01* | USA |
| 61 | Venous thromboembolism | I82.9 | Thrombo inCode^®^ | Spain, France |
| 62 | Colorectal cancer | C18.9 | *KRAS, BRAF* | Switzerland |
| 63 | Non–small cell lung cancer | C34.9 | *EGFR* | USA |
| 64 | Acquired immune deficiency syndrome (AIDS) | B24 | *HLA-B*57:01* | USA |
| 65 | Colorectal cancer (Familial adenomatous polyposis) | D12.6 | *APC* | Spain |
| 66 | Spinal muscular atrophy | G12.9 | *SMN1* | USA |
| 67 | Inflammatory bowel disease | K50, K51, K52 | *TPMT* | New Zealand |
| 68 | Epilepsy | G40.9 | *HLA-B*15:02* | Thailand |
| 69 | Inflammatory disease | K50, K51, K52, M05, K20.9 | *TPMT* | United Kingdom |
| 70 | Colorectal cancer (Lynch syndrome) | C18.9 | MMR genes | Singapore |
| 71 | Colorectal cancer, Polyposis syndrome | C18.9 | *BMPR1A, SMAD4, APC, MUTYH,*  *TP53, CDH1, STK11, PTEN,*  *MLH1, MSH2, MSH6, PMS2* | USA |
| 72 | Atrial fibrillation | I48 | *VKORC1, CYP2C9* | USA |
| 73 | Breast cancer,  Ovarian cancer | C50.9, C56.9 | *BRCA1/2* | United Kingdom |
| 74 | Atrial fibrillation | I48 | *VKORC1, CYP2C9* | United Kingdom, Sweden |
| 75 | Atrial fibrillation | I48 | *VKORC1, CYP2C9* | The Netherlands |
| 76 | Atrial fibrillation | I48 | *VKORC1, CYP2C9* | The Netherlands |
| 77 | Atrial fibrillation | I48 | *VKORC1, CYP2C9* | Croatia |
| 78 | Neovascular macular degeneration | H35.3 | *CFH, ARMS2/HTRA1, C3, C2, CFB* | USA |
| 79 | Myocardial infarction | I21.9 | *CYP2C19* | Serbia |
| 80 | Rheumatoid arthritis | Μ05 | *MTHFR* | South Korea |
| 81 | Chronic nephropathies | I51.6, N19 | *ACE* | Switzerland |
| 82 | Colorectal cancer (MAP syndrome) | C18.9 | *MUTYH* | The Netherlands |
| 83 | Breast cancer,  Ovarian cancer | C50.9, C56.9 | *BRCA1/2* | United Kingdom |
| 84 | Coeliac disease | K90.0 | *HLA-DQ2,5, HLA-DQ8* | The Netherlands |
| 85 | Acute coronary syndrome | I25.9 | *CYP2C19* | USA |
| 86 | Adenocarcinoma of the lung | C34.9 | *EGFR* | USA |
| 87 | Neurofibromatosis type 1 | Q85.01 | *SPRED1, NF1* | USA |
| 88 | Schizophrenia | F20 | *CYP2D6, CYP2C19* | Denmark |
| 89 | Diabetes type 2 | E11 | *HNF1A, HNF4A, GCK* | USA |
| 90 | Atrial fibrillation | I48 | *VKORC1, CYP2C9* | USA |
| 91 | Colorectal cancer (Lynch syndrome) | C18.9 | *MLH1, MSH2, MSH6, PMS2* | USA |
| 92 | Cystic fibrosis | Ε84.9 | *CFTR* | United Kingdom |
| 93 | Breast cancer | C50.9 | *HER-2* | Switzerland |
| 94 | Breast cancer (Lynch syndrome) | C18.9 | *MLH1, MSH2, MSH6, PMS2, BRAF* | USA |
| 95 | X-linked hemophilia | D66 | Factor VIII *(F8),* factor IX *(F9)* | China (Taiwan) |
| 96 | Cystic fibrosis | Ε84.9 | *CFTR* | Australia |

| Supplementary Table 4. Cost of genomic testing and economic evaluation of interventions | | | | | | | | | | | | | | | | | | |  |
| --- | --- | --- | --- | --- | --- | --- | --- | --- | --- | --- | --- | --- | --- | --- | --- | --- | --- | --- | --- |
| r/n | **Gene/Genomic test** | **Cost of PGx** | | **Cost of intervention w/o PGx** | | | | | | **Cost of intervention w PGx** | | **ICER** | | | | | **ICUR** | **Monetary value (year)** |  |
| 1 | *LDLR, APOB* | $3,480 | | $10,396 | | | | | | $15,594 | |  | | | | | $519,813/QALY | 2013 |  |
| 2 | *EGFR* | SG$380 | | SG$47,100 | | | | | | SG$44,700 | |  | | | | | **Dominant** | 2010 |  |
| 3 | *TPMT* | Can$459.63 | | Can$654 | | | | | | Can$1,090 | | Not calculated | | | | | Not calculated | 2008 |  |
| 4 | *KRAS* | ¥20,000 ($220) | | ¥3,160,000  ($35,000) | | | | | | ¥2,600,000  ($29,000) | | **Dominant** | | | | | **Dominant** | 2010 |  |
| 5 | HPV DNA | R200 ($30) | | R91,767 | | | | | | R92,557 | |  | | | | | R25,414/QALY | 2006 |  |
| 6 | *CFTR* | €166^a^ | | €30,077,000 | | | | | | €30,349,000 | | €29,200/LY | | | | |  | 2009 |  |
|  |  | €417^b^ | |  |  |  |  |  |  |  |  |  |  |  |  |  |  |  |  |
| 7 | *CYP2C19* | $500 | | $15,800 | | | | | | $14,900 | |  | | | | | **Dominant** | 2010 |  |
| 8 | MammaPrint^®^ | €2,675 | | €15,904 | | | | | | €16,989 | | | €1,257/LY | | | €1,457/QALY | | 2013 |  |
|  | Oncotype DX^®^ | €3,200 | |  |  |  |  |  |  | €17,869 | | | **Dominant** | | | **Dominant** | |  |  |
| 9 | *KCNQ1, HERG, SCN5A, ANK2, KCNE1* | $5,400^c^,  $900^d^ | | $16,048 | | | | | | $25,467 | | Dominated | | | | | $67,400/QALY | 2008 |  |
| 10 | *LDLR* | €425 | | €4,298 | | | | | | €8,891 | | €3,423/LY | | | | |  | 2005 |  |
| 11 | *KCNJ11* | $705 | | $71,784 | | | | | | $59,256 | |  | | | | | **Dominant** | 2008 |  |
|  | *ABCC8* | $2,110 | |  |  |  |  |  |  |  |  |  |  |  |  |  |  |  |  |
| 12 | *MYH7, MYBPC3, TNNT2, TNNI3* | €552^e^,  €225^f^ | | €14,872^g^ | | | | | €19,459^g^ | | | | | €14,397/LY^g^ | | |  | 2007 |  |
|  |  |  |  | €7,326^h^ | | | | | €9,867^h^ | | | | | €16,185/LY^h^ | | |  |  |  |
| 13 | *FGFR2, TOX3, MAP3K1,* rs13387042, rs13281615,*FGF10, LSP1-H19* | $945 | | $629,297,039 | | | | | | $870,639,365 | |  | | | | | $634,133/QALY | 2012 |  |
| 14 | *UGT1A1*28* | €71 | | €1,284.60 | | | | | | €1,383.70 | | €942,8-€1,090.1/febrile neutropenia avoided | | | | |  | 2006 |  |
| 15 | 4q25 chromosome | $50 - $200 | | NA | | | | | | NA | |  | | | | | $47,148/QALY - **Dominant**  (according to adherence rate) | 2005 |  |
| 16 | Oncotype DX^®^ | ¥35,0000  ($3,500) | | ¥1,237,698 | | | | | | ¥1,391,263 | |  | | | | | ¥636,752 ($6,368)/QALY | 2013 |  |
| 17 | SNPs for RA | €750 | | €16,784 | | | | | | €17,611 | |  | | | | | €57,606/QALY | 2014 |  |
| 18 | HPV-16/18 DNA | $48.24 | | $1,230 | | | | | | $1,389 | |  | | | | | $6,910/QALY | 2013 |  |
| 19 | *LDLR, APOB* | Α$1,512 | | A$704,745 | | | | | | A$80,8377 | | A$4,154/YoLS | | | | | A$3,565/QALY | 2013 |  |
| 20 | *HLA-B*15:02* | $192.20 | | $164 | | | | | | $205 | |  | | | | | $85,697/QALY | ΝΑ |  |
| 21 | *KRAS* | $224 | | | | $34,291 | | | | | $57,348 | | | | $2,814,338/YoLS | |  | 2010 |  |
|  | *KRAS+BRAF* | $303 | | | |  |  |  |  |  | $56,324 | | | | $648,396/YoLS | |  |  |  |
| 22 | *HLA-B*15:02* | $270 | | $4,110 | | | | | | $4,680 | |  | | | | | $29,750/QALY | 2010 |  |
| 23 | *HLA-B*58:01* | $270 | | $4,194 | | | | | | $4,419 | |  | | | | | Dominated | 2012 |  |
| 24 | *DPYD*2A* | €75 | | €2,817 [$3,828] | | | | | | €2,772 [$3,767] | |  | | | | |  | 2014 |  |
| 25 | *MLH1, MSH2, MSH6, PMS2, BRAF* | €109,305 (MLH1/PMS2 or MSH2/MSH6), €3,400 (BRAF) | | €218,581,280 | | | | | | €242,028,209 | | €77,268/LY | | | | |  | 2012 |  |
| 26 | BREVAGenTM^®^ | $945 | |  | | | | | | NA | | $105,000/YoLS | | | | | $51,000/QALY | 2012 |  |
| 27 | *TPMT* | $300 | | $9,691 | | | | | | $15,818 | |  | | | | | $49,156/QALY | 2007 |  |
| 28 | HCM genes | Α$2,000 | |  | | | | | | NA | | A$12,720/LY | | | | | A$785/QALY | NA |  |
| 29 | *BRAF* | $97.45 | | $2,7084.48 | | | | | | $28,213.49 | |  | | | | | $33,96/QALY | 2010 |  |
| 30 | *EGFR* | ¥21,000 [$201,9] | | ¥5,130,000 [$49,400] | | | | | | ¥5,470,000 [$52,600] | |  | | | | | ¥3,380,000 [$32500000]/QALY | 2012 |  |
| 31 | *EGFR, ALK* | $201 (EGFR), $136  (ALK ICH),$489  (ALK FISH) | | $82,762 | | | | | | $83,413 | | $102,000/LY | | | | | $136,000/QALY | 2013 |  |
| 32 | *UGT1A1*28* | $107 | | $475,800 | | | | | | $475,910 | |  | | | | | $2,058,200/QALY | 2009 |  |
| 33 | *CYP2C19* | NZ$175 | | NZ$85,342 (prasugrel) | | | | | | NZ$84,646 | |  | | | | | **Dominant** | 2009 |  |
|  |  |  |  | NZ$84,171 (clopidogrel) | | | | | |  |  |  |  |  |  |  | NZ$24,617/QALY |  |  |
| 34 | *KIF6* | $100 | | $31,674 | | | | | | $37,085 | | $177,927/event avoided | | | | | $45,017/QALY | 2010, 2012 |  |
|  |  |  |  |  |  |  |  |  |  |  |  | $40,687/YoLS | | | | |  |  |  |
| 35 | *CYP2C19* | $300 | | $19,147 | | | | | | $19,231 | |  | | | | | $4,200/QALY | 2011 |  |
| 36 | *BRCA1/2* | $2,542 | | $117,000 | | | | | | $118,000 | |  | | | | | $9,000/QALY | 2006 |  |
| 37 | Factor V Leiden, Factor II | €100,50 | | €32,823 | | | | | | €111,159 | |  | | | | | €171,584.3/QALY | 2009 |  |
| 38 | *HLA-A*31:01* | £90.40 | | £10,508 | | | | | | £10,808 | | Dominated | | | | | £12,808/QALY | 2010,2011 |  |
| 39 | *HLA-B*58:01* | THB1,000,000 | | THB228,806,808.89 | | | | | | THB229,730,727.91 | |  | | | | | THB156,937.04/QALY | 2013 |  |
| 40 | *MLH1, MSH2, MSH6, PMS2, BRAF* | $999.6  (MLH1, MSH2, MSH6, PMS2),  $99.96 (BRAF) | | $4,032 | | | | | | $5,735 | | $6,025/LY | | | | |  | 2012 |  |
| 41 | Corus CAD^®^ | $1,245 | | $0 | | | | | | $3,240,000 | |  | | | | |  | 2012 |  |
| 42 | *LPA* | $150 | | NA | | | | | | $3,076,521 | | $30,846/  CVD event prevented | | | | | $24,942/QALY | 2009 |  |
| 43 | *PTEN* | $11,425  (per mutation) | | $0 | | | | | | NA | |  | | | | | $58,884-$107,390/QALY (male) | 2014 |  |
|  |  |  |  |  |  |  |  |  |  |  |  |  |  |  |  |  | $50,569-$155,367/QALY (female) |  |  |
| 44 | *KRAS* | $243 | | | $48,576 | | | $35,075 | | | |  | | | | | Less costly,  same effectiveness | 2009 |  |
|  |  | €280 (Germany) | | | €35,852 | | | €26,292 | | | |  |  |  |  |  |  |  |  |
| 45 | *HLA-B*15:02* | THB3,000 | | THB398,549.94 | | | | | | THB300,000 | |  | | | | |  | NA |  |
| 46 | *BRCA1/2* | £306 | | £96,833,471 | | | | | | £99,894,892 | |  | | | | | £4,339/QALY | 2014-2015 |  |
| 47 | *CYP2C19* | $200 | | $76,906 | | | | | | $76,450 | |  | | | | | **Dominant** | 2016 |  |
| 48 | *HLA-B*58:01* | NT$2,648 | | NT$39,419 | | | | | | NT$42,040 | |  | | | | | NT$234,610.94/QALY | 2015 |  |
| 49 | *BRCA1/2,* | | $2,178 | $23,954 (BRCA1/2) | | | | | | $24,231 (7-gene) | |  | | | | | $48,328/QALY | 2015 |  |
|  | 7-gene  (*PALB2, TP53, CDH1, STK11, PTEN, BRCA1/2*) | | $2,418 |  |  |  |  |  |  |  |  |  |  |  |  |  |  |  |  |
| 50 | *MLH1, MSH2, MSH6, PMS2, BRAF* | $808 (MLH1), $683 (MSH2), $983 (MSH6), $983 (PMS2),  $62 (BRAF) | | ΝΑ | | | | | | $23,026,000 | | $22,552/LY | | | | |  | 2007 |  |
| 51 | GEN test | €2,549 | | €12,688 | | | | | | €10,183 | |  | | | | |  | ΝΑ |  |
| 52 | *KRAS* | €321 | | ΝΑ | | | | | | ΝΑ | |  | | | | |  | NA |  |
| 53 | MammaPrint^®^ | $4,200 | | $21,598 (MammaPrint^®^) | | | | | | $27,882  (Oncotype DX^®^) | |  | | | | | **Dominant** | 2009 |  |
|  | Oncotype DX^®^ | $3,975 | |  |  |  |  |  |  |  |  |  |  |  |  |  |  |  |  |
| 54 | *UGT1A1*28* | $102.83 | | $13,058 | | | | | | $12,786 | |  | | | | | **Dominant** | 2007 |  |
| 55 | *UGT1A1*28* | $375 | | ΝΑ | | | | | | NA | | **Dominant**(Caucasian, African) | | | | |  | 2006 |  |
|  |  |  |  |  |  |  |  |  |  |  |  | $95,455/severe neutropenia avoided (Asian) | | | | |  |  |  |
|  |  |  |  |  |  |  |  |  |  |  |  | $6,818,203/LY (Asian) | | | | |  |  |  |
| 56 | *VKORC1, CYP2C9* | £20.00 | | £5,880 | | | | | | £5,921 | |  | | | | | £13,226/QALY | 2011 |  |
| 57 | *APOE* (*ε4* allele) | Can$325 | | Can$131,090 | | | | | | Can$132,105 | |  | | | | | Can$38,016/QALY | 2009 |  |
| 58 | *HLA-B*57:01* | €29–€183 | | €1,322 | | | | | | €1,344 | | €630.16/hypersensitivity reaction avoided | | | | |  | 2008 |  |
| 59 | Oncotype DX^®^ | £2,576 | | £22,270 | | | | | | £23,130 | |  | | | | | £5,529/QALY | 2011 |  |
| 60 | *HLA-B*57:01* | $68 | | $472,210 | | | | | | $472,320 | |  | | | | | $36,700/QALY | 2006 |  |
| 61 | Thrombo inCode^®^ | €180 | | €2795.61^i^ | | | €832.58^i^ | | | | |  | | | | | **Dominant** | 2013 |  |
|  |  |  |  | €1366.30^j^ | | | €848.38^j^ | | | | |  |  |  |  |  |  |  |  |
| 62 | *KRAS, BRAF* | €394 | | €3,983 | | | | | | €34,771 | |  | | | | | €62,653/QALY | 2010 |  |
| 63 | *EGFR* | $320 | | $57,238 | | | | | | $66,447 | | $78,367/LY | | | | | $162,018/QALY | 2006 |  |
| 64 | *HLA-B*57:01* | $87.92 | | $3,067.43 | | | | | | $3,084.73 | | $328.32/hypersensitivity reaction avoided | | | | |  | 2007 |  |
| 65 | *APC* | €1,164.90 | | €13,928.82 | | | | | | €8,038.93 | |  | | | | | **Dominant** | 2005 |  |
| 66 | *SMN1* | $425 | | $4,714,165 | | | | | | $44,295,289 | | $4,985,028/spinal muscular atrophy avoided | | | | | $4,889,675/QALY | 2009 |  |
| 67 | *TPMT* | NZ$120,000 ($78,000) | | ΝΑ | | | | | | NA | |  | | | | |  | 2004 |  |
| 68 | *HLA-B*15:02* | THB1,000 | | THB42,000^k^ | | | THB50,000^k^ | | | | |  | | | | | THB222,000/QALY^k^ | 2011 |  |
|  |  |  |  | ΤΗΒ19,000^l^ | | | THB23,000^l^ | | | | |  |  |  |  |  | THB130,000/QALY^l^ |  |  |
| 69 | *TPMT* | £20 | | £1,966.78 | | | | | | £1,683.40 | |  | | | | |  | 2009-2010 |  |
| 70 | MMR genes | SG$1,446 | | SG$33,625 | | | | | | SG$20,037 | | **Dominant** | | | | |  | 2010 |  |
| 71 | *BMPR1A , SMAD4, APC, MUTYH, TP53, CDH1, STK11, PTEN, MLH1, MSH2, MSH6, PMS2*  (NGS panel) | $2,700 | | NA | | | | | | NA | | $122,316/LY | | | | | $144,235/QALY | 2014 |  |
| 72 | *VKORC1, CYP2C9* | $175 | | $46,808 | | | | | | $46,970 | |  | | | | | $60,750/QALY | 2007 |  |
| 73 | *BRCA1/2* | £540 | | £960.59 | | | | | | £1,630.96 | |  | | | | |  | 2013 |  |
| 74 | *VKORC1, CYP2C9* | £35.03 | | | £8,614 | | | £8,640 | | | |  | | | | | £6,702/QALY | 2014 |  |
|  |  | 440 SEK | | | 88,072 SEK | | | 88,453 SEK | | | |  |  |  |  |  | 253,848SEK/QALY |  |  |
| 75 | *VKORC1, CYP2C9* | €40 | | €7,934.01 | | | | | | €7,949.16 | |  | | | | | €2,658/QALY | 2011 |  |
| 76 | *VKORC1, CYP2C9* | €40 | | €9,644^m^ | | | €9,677^m^ | | | | |  | | | | | €28,349/QALY^m^ | 2012 |  |
|  |  |  |  | €9,616^n^ | | | €9,649^n^ | | | | |  |  |  |  |  | €24,427/QALY^n^ |  |  |
| 77 | *VKORC1, CYP2C9* | €140,25 | | €219.70 | | | | | | €538.70 | |  | | | | | €31,225/QALY | NA |  |
| 78 | *CFH, ARMS2/HTRA1, C3, C2, CFB* | $1,461 | | NA | | | | | | NA | |  | | | | | **Dominant** | 2012 |  |
| 79 | *CYP2C19* | €63 | | NA | | | | | | €2,547 (*CYP2C19*1/*1*) | |  | | | | |  | NA |  |
|  |  |  |  |  |  |  |  |  |  | €2,799 (*CYP2C19*1/*2*,*CYP2C19*2/*2*) | |  |  |  |  |  |  |  |  |
| 80 | *MTHFR* | Won60,000($50) | | Won851,415 ($710) | | | | | | Won788,664($658) | |  | | | | |  | NA |  |
| 81 | *ACE* | €49 | | €43,905 | | | | | | €42,837 | | **Dominant** | | | | |  | 2005 |  |
| 82 | *MUTYH* | €645 | | ΝΑ | | | | | | NA | | €42,000/LY | | | | | €25,000/QALY | 2006 |  |
| 83 | *BRCA1/2* | £50 | | £1,741 | | | | | | £1,677 | | £2,079/LY | | | | |  | 2010 |  |
| 84 | *HLA-DQ2,5,*  *HLA-DQ8* | €18,357^o^ | | €20,073 | | | | | | €35,875 | |  | | | | |  | 2013 |  |
| 85 | *CYP2C19* | $315 | | $8,866,274 | | | | | | $8,421,422 | |  | | | | |  | 2012 |  |
| 86 | *EGFR* | $243 | | $29,987 | | | | | | $36,460 | |  | | | | | $110,658/QALY | 2009 |  |
| 87 | *SPRED1, NF1* | $600 (SPRED1), $1,200 (NF1) | | $5,774 | | | | | | $5,821 | |  | | | | |  | 2011 |  |
| 88 | *CYP2D6, CYP2C19* | DKK1,195 (US$214) | | DKK153,536 (US$27,350) | | | | | | DKK 131,141 (US$23,361) | |  | | | | |  | 2010 |  |
| 89 | *HNF1A, HNF4A, GCK* | $2,580 | | $44,400 | | | | | | $46,800 | |  | | | | | $205,000/QALY | 2011 |  |
| 90 | *VKORC1, CYP2C9* | $500-$650 | | NA | | | | | | NA | |  | | | | | <$50,000/QALY | 2007 |  |
| 91 | *MLH1, MSH2, MSH6, PMS2* | $3,495  (All genes),  $860 (MLH1),  $771 (MSH2),  $933 (MSH6),  $884 (PMS2) | | NA | | | | | | NA | |  | | | | | $26,000/QALY | 2009 |  |
| 92 | *CFTR* | £786 | | £48,160 | | | | | | £57,185 | |  | | | | |  | 2012-2013 |  |
| 93 | *ERBB2* (HER-2) | €53 (IHC),  €686 (FISH) | | €32,258 | | | | | | €38,215 | |  | | | | | €12,245/QALY | 2009 |  |
| 94 | *MLH1, MSH2, MSH6, PMS2, BRAF* | $880(per MMR gene),  $110(BRAF testing),  $280(IHC BRAF),  $490(MSI BRAF) | | $11,242 | | | | | | $23,642 | | $117,000/LY | | | | |  | 2010 |  |
| 95 | Factor VIII *(F8),* factor XI *(F9)* | NT$4,500-NT$25,000 | | NT$414,433,180^p^ (hemophilia care) | | | | | | NT$85,918,250^q^ (genetic testing) + ΝΤ$211,445,500^p^ (hemophilia care) | | NT$1,219,473/LY | | | | |  | 2009 |  |
| 96 | *CFTR* | A$116.77 | | A$11,500,000 | | | | | | A$9,000,000 | | **Dominant** | | | | |  | 2010 |  |

**a**: Cost of test including immunoreactive trypsinogen (IRT) and DNA testing, b: Cost of DNA sequencing, **c**: Genetic testing for a 10-year-old, first-degree family member of an index case (proband), **d**: Genetic testing for first-degree relatives, if a mutation in the index case was found, **e**: Genetic diagnostic test of proband, **f**: Genetic diagnostic test of siblings, **g**: Whole family scenario, **h**: One child scenario, **i**: S. PAU case-study population, **j**: MARTHA case-study population, **k**: Epilepsy treatment model, **l**: Neuropathic pain model, **m**: Intervention including phenprocoumon treatment, **n**: Intervention including acenocoumarol treatment, **o**: Including testing for 110 children, **p**: Total hemophilia care costs, **q**: Total genetic testing costs

| Supplementary Table 5A. Incremental cost-utility ratios (ICUR) of “cost-effective” individualized interventions | | | | | |
| --- | --- | --- | --- | --- | --- |
| Disease | Gene/  Genomic Test | ICUR | Suggested willingness to pay-threshold | Country | Reference |
| Breast cancer | MammaPrint^®^ | €1,457/QALY | €30,000/QALY | Spain | [*8*] |
|  | Oncotype DX^®^ | ¥636,752/QALY | ¥6,000,000/QALY | Japan | [*16*] |
|  | Oncotype DX^®^ | £5,529/QALY | £20,000-£30,000/QALY | UK | [*59*] |
|  | *BRCA1/2* | £4,339/QALY | £20,000/QALY | UK | [*46*] |
|  | *BRCA1/2* | $9,000/QALY | $50,000/QALY | USA | [*36*] |
|  | *PALB2,TP53,CDH1,STK11,PTEN,BRCA1/2* | $48,328/QALY^+^ | $100,000/QALY | USA | [*49*] |
| Non-small cell lung cancer | *EGFR* | ¥3,380,000/QALY | ¥5,000,000-¥6,000,000/QALY | Japan | [*30*] |
|  | *EGFR, ALK* | $136,000/QALY | $200,000/QALY | USA | [*31*] |
| Familial hypercholesterolemia | *LDLR, APOB* | A$3,565/QALY | A$6,000/QALY | Australia | [*19*] |
| Hypertrophic cardiomyopathy | HCMgenes | A$785/QALY | A$50,000/QALY |  | [*28*] |
| Epilepsy | *HLA-B*15:02* | $29,750/QALY | $50,000/QALY | Singapore* | [*22*] |
|  | *HLA-A*31:01* | £12,808/QALY | £20,000-£30,000/QALY | UK | [*38*] |
| Idiopathic pulmonary fibrosis | *TPMT* | $49,156/QALY | $50,000/QALY | USA | [*27*] |
| Papillary thyroid cancer | *BRAF* | $33.96/QALY | $50,000/QALY | USA | [*29*] |
| Acute coronary syndrome | *KIF6* | $45,017/QALY | $50,000-$100,000/QALY | USA | [*34*] |
|  | *CYP2C19* | $4,200/QALY | $100,000/QALY | USA | [*35*] |
|  | *CYP2C19* | NZ$24,617/QALY | NZ$50,000/QALY | New Zealand | [*33*] |
| Gout | *HLA-B*58:01* | THB156,937.04/QALY | THB160,000/QALY | Thailand | [*39*] |
| Cardiovascular disease | *LPA* | $24,942/QALY | $50,000/QALY | USA | [*42*] |
| Atrial fibrillation | *VKORC1, CYP2C9* | £13,226/QALY | £20,000-£30,000/QALY | UK | [*56*] |
|  |  | £6,702/QALY | £20,000-£30,000/QALY | UK | [*74*] |
|  |  | 253,848SEK/QALY | 500,000 SEK/QALY |  | [*74*] |
|  |  | €2,658/QALY | €20,000/QALY | The Netherlands | [*75*] |
|  |  | €31,225/QALY | €30,000-€40,000/QALY | Croatia | [*77*] |
|  | 4q25chromosome | $47,148/QALY | $50,000/QALY | USA | [*15*] |
| HIV | *HLA-B*57:01* | $36,700/QALY | $50,000-$100,000/QALY | USA | [*60*] |
| MAP syndrome | *MUTYH* | €25,000/QALY | €80,000/QALY | The Netherlands | [*82*] |
| Colorectal cancer | *MLH1, MSH2,*  *MSH6, PMS2* | $26,000/QALY | $50,000/QALY | USA | [*91*] |
| Amnestic mild cognitive impairment | (*APOE*) *ε4*allele | Can$38,016/QALY | Can$20,000-Can$50,000-Can$100,000/QALY | Canada | [*57*] |

+In comparison with testing only for *BRCA1* and *BRCA2* genes.

| Supplementary Table 5B. Incremental cost-utility ratios (ICUR) of “non-cost-effective” individualized interventions | | | | | |
| --- | --- | --- | --- | --- | --- |
| Disease | Gene/  Genomic Test | ICUR | Suggested willingness to pay-threshold | Country | Reference |
| Familial hypercholesterolemia | *LDLR,APOB* | $519,813/QALY | $150,000/QALY | USA | [*01*] |
| Long-QT syndrome | *KCNQ1, HERG,*  *SCN5A, ANK2,*  *KCNE1* | $67,400/QALY | $50,000/QALY | USA | [*09*] |
| Breast cancer | *FGFR2,TOX3,MAP3K1,* rs13387042, rs13281615,*FGF10, LSP1-H19* | $634,133/QALY | $50,000/QALY | USA | [*13*] |
|  | BREVAGenTM^®^ | $51,000/QALY | $50,000/QALY^+^ | USA | [*26*] |
| Rheumatoid arthritis | SNPs | €57,606/QALY | €20,000/QALY | The Netherlands | [*17*] |
| Epilepsy | *HLA-B*1502* | $85,697/QALY | $50,000/QALY | China* | [*20*] |
|  |  | THB222,000/QALY | THB120,000/QALY | Thailand | [*68*] |
| Neuropathic pain |  | THB130,000/QALY |  |  | [*68*] |
| HIV | *UGT1A1*28* | $2,058,200/QALY | $100,000/QALY | USA | [*32*] |
| Venous Thromboembolism | Factor V Leiden, Factor II | €171,584.3/QALY | €40,000-€50,000/QALY | Italy | [*37*] |
| Cowden syndrome | *PTEN* | $58,884-$107,390/QALY (male) | $100,000/QALY^++^ | USA | [*43*] |
|  |  | $50,569-$155,367/QALY (female) |  |  | [*43*] |
| Non–small cell lung cancer | *EGFR* | $162,018/QALY | $150,000/QALY | USA | [*63*] |
| Adenocarcinoma of the lung | *EGFR* | $110,658/QALY | $100,000/QALY | USA | [*86*] |
| Spinal muscular atrophy | *SMN1* | $4,889,675/QALY | $50,000-$100,000/QALY | USA | [*66*] |
| Colorectal cancer | *BMPR1A,SMAD4, APC,MUTYH, TP53,CDH1, STK11,PTEN, MLH1,MSH2, MSH6,PMS2* | $144,235/QALY | $100,000/QALY | USA | [*71*] |
| Atrial fibrillation | *VKORC1, CYP2C9* | $60,750/QALY | $50,000/QALY | USA | [*72*] |
|  |  | €28,349/QALY (Phenprocoumon) | €20,000/QALY | The Netherlands | [*76*] |
|  |  | €24,427/QALY (Acenocoumarol) |  |  | [*76*] |
| Type 2 diabetes | *HNF1A, HNF4A,*  *GCK* | $205,000/QALY | $50,000/QALY | USA | [*89*] |

^*^The currency used is different from the currency of the country in which the corresponding economic evaluation study was carried out. Researchers chose US willingness to pay thresholds, expressing costs in US $ ($).

^++^Incremental cost-utility ratio varies depending upon age and gender. As a result, using the $100000/QALY threshold, testing strategy may be cost-effective under specific circumstances.
